# Knockout of thyroid hormone receptor alpha a (*thraa*) enhances cardiac regeneration in zebrafish through metabolic and hypoxic regulation

**DOI:** 10.1186/s12964-025-02350-5

**Published:** 2025-07-16

**Authors:** Man Yee Cheung, Chunmei Jiang, Imtiaz Ul Hassan, Hui Wang, Donghao Guo, Daniel Wuyang Dio, Huan Yan, Jianmin Sun, Xufeng Qi, Dongqing Cai, Wei Ge, Sheue-yann Cheng, Wai-Yee Chan, Hui Zhao

**Affiliations:** 1 Key Laboratory for Regenerative Medicine, School of Biomedical Sciences, Faculty of Medicine, Ministry of Education, The Chinese University of Hong Kong, New Territories, Hong Kong S.A.R., China; 2 Translational and Functional Genomics Branch, National Human Genome Research Institute, Bethesda, MD 20892 USA; 3 Division of Cardiology, Renji Hospital, School of Medicine, Shanghai Jiao Tong University, Shanghai, China; 4 Department of Pathogen Biology and Immunology, School of Basic Medical Sciences, Ningxia Medical University, No. 1160 Shengli Street, Yinchuan, 750004 China; 5 Key Laboratory of Regenerative Medicine of Ministry of Education, Department of Developmental & Regenerative Biology, Jinan University, Guangzhou, 510632 Guangdong China; 6 Department of Biomedical Sciences and Centre of Reproduction, Development and Aging (CRDA), Faculty of Health Sciences, University of Macau, Taipa, 999078 Macau China; 7 Laboratory of Molecular Biology, National Cancer Institute, National Institutes of Health, Bethesda, MD 20892 USA; 8 Joint Laboratory of Bioresources and Molecular Research of Common Diseases, Kunming Institute of Zoology - The Chinese University of Hong Kong (KIZ-CUHK), The Chinese University of Hong Kong, New Territories, Hong Kong S.A.R., China; 9 Hong Kong Branch of CAS Center for Excellence in Animal Evolution and Genetics, The Chinese University of Hong Kong, New Territories, Hong Kong S.A.R., China

**Keywords:** Thyroid hormone, Metabolism, Hypoxia, Heart regeneration, Inflammation, Cardiomyocytes, Zebrafish

## Abstract

**Background:**

Thyroid hormone (TH) signaling drives cardiomyocyte (CM) maturation in endothermic animals. Elevated TH levels, coupled with increased basal metabolism, promote CM cell cycle exit and polyploidization, thus limiting heart regenerative potential. However, a comprehensive understanding of TH and its receptors, thyroid hormone receptors (TRs), orchestrating with other regulatory processes for heart regeneration, such as the hypoxia signaling pathway and post-injury metabolic switches, remains elusive.

**Results:**

Here, we investigated the molecular mechanisms of TH signaling in heart regeneration using a time-course sequencing experiment. We assessed heart regeneration capacity in *thyroid hormone receptor alpha a* (*thraa*) mutant zebrafish, which carry an 8-bp insertion that leads to truncation of the *Thraa* protein and impaired TH signaling. The *thraa* + 8 bp mutant zebrafish exhibited an enhanced heart regenerative response. Our study showed that, in *thraa*^+/–^ mutants, a transiently augmented inflammatory response and an extended CM proliferative window are associated with metabolic switches across different phases. Moreover, we found that *thraa* transcriptionally regulates *hypoxia-inducible factor 3 subunit alpha* (*hif3a*), and its knockout in zebrafish impairs heart regeneration.

**Conclusions:**

In conclusion, our study highlights the role of TH signaling via *thraa* in modulating zebrafish heart regeneration through metabolic regulation, inflammation, cardiac tissue regeneration, and its interplay with *hif3a*.

**Supplementary Information:**

The online version contains supplementary material available at 10.1186/s12964-025-02350-5.

## Introduction

The limited regenerative capacity of the adult human heart results in irreversible damage following ischemic heart disease (IHD). Acute myocardial infarction (AMI) or myocardial injury leads to adverse cardiac remodeling and heart failure due to the heart’s restricted regenerative potential. During long-term cardiac remodeling, persistent inflammation, pathological hypertrophy in cardiomyocytes (CMs), and unresolved fibrosis are commonly observed in the failing heart. Common complications in patients with AMI include impaired diastolic and systolic function, an increased risk of arrhythmias, and post-infarction pericarditis [1].

Interestingly, cardiac regenerative capacity has been shown to be developmentally regulated [2]. Several studies have reported that mammalian species, including humans, exhibit a transient ability to regenerate damaged heart tissue during fetal and early neonatal stages, but this regenerative potential rapidly diminishes after birth [3–10]. The mouse model (*M. musculus*) has been extensively studied and demonstrates a well-defined regenerative window for cardiac tissue repair following ischemic injury, with this regenerative potential lost by postnatal day 7 [3, 11, 12]. In contrast, some non-mammalian vertebrates, such as amphibians and teleost fish, retain robust cardiac regenerative abilities throughout adulthood. Axolotl (*A. mexicanum*), Iberian ribbed newt (*P. waltl*), eastern newt (*N. viridescens*), and zebrafish (*D. rerio*) are capable of regenerating heart tissue without scarring after ventricular resection or cryoinjury [13–17]. Interestingly, some species exhibit distinct regenerative potential compared to their evolutionary relatives. For example, the western clawed frog (*X. tropicalis*) and surface-dwelling form of Mexican tetra (*A. mexicanus*) demonstrate regenerative potential in the adult stage, while the African clawed frog (*X. laevis*), blind cave form of Mexican tetra (*A. mexicanus*), and Medaka Japanese rice fish (*O. latipes*) exhibit limited or incomplete heart regenerative potential [18–22].

Emerging evidence suggests that TH signaling plays a critical role in heart regeneration, indicating that the loss of regenerative capacity in adult mammals is an evolutionary trade-off associated with endothermy [23]. During development, increasing circulating TH levels and TH-dependent metabolic rates enable thermoregulation in endothermic species, allowing them to maintain a constant body temperature and adapt to their habitats. Meanwhile, this hormonal shift rewires mitochondrial function and myocardial energy metabolism, resulting in terminal differentiation, polyploidization, and permanent exit from cell cycle in CMs, processes that impair heart regeneration. These effects on CM phenotype indicate that TH signaling plays a pivotal role in the transition from a regenerative to a non-regenerative state. Numerous studies have also reported a strong correlation between myocardial metabolic state and regenerative capacity [24–35]. In particular, metabolic reprogramming, characterized by a shift from oxidative phosphorylation (OXPHOS) to glycolysis, is a key hallmark of heart regeneration, reflecting the metabolic characteristics of early development and tissue repair [36, 37].

In addition to TH, hypoxia regulates metabolic reprogramming in the ischemic myocardium, where oxygen supply is limited [38, 39]. Hypoxia-inducible factors (HIFs), a family of transcription factors, are activated to trigger an adaptive metabolic response under hypoxic conditions, mitigating reactive oxygen species (ROS) production and protecting cells from oxidative stress. Specifically, HIF1α, upon activation, dimerizes with HIF1β to mediate metabolic adaptation by promoting a transition from oxidative to glycolytic metabolism, altering mitochondrial function, and selectively utilizing metabolic substrates [40, 41]. Many metabolic genes have been identified as HIF1 targets, including glucose transporter protein type 1 (GLUT1), hexokinase 2 (HK2), phosphofructokinase-1 (PFK1), and pyruvate kinase M2 (PKM2) [42]. Additionally, HIF3α, another member of the HIF protein family, also exhibits oxygen-dependent transcriptional activity [43]. In zebrafish, Hif3a has been demonstrated to share similar metabolic transcriptional targets with Hif1a [44], suggesting their coordinated roles in cardiac repair and regeneration through metabolism.

Despite the established roles of TH signaling, hypoxia, and metabolic reprogramming in heart regeneration, the molecular mechanisms orchestrating their interplay, particularly through thyroid hormone receptor alpha (TRα), remain largely unexplored. The active form of TH, triiodothyronine (T3), binds to nuclear thyroid hormone receptors (TRs), which regulate gene expression via thyroid hormone response elements (TREs). Previous research has shown that ligand-bound TRα transcriptionally regulates mitochondrial genes in mouse hearts, highlighting the role of TH/TRα signaling axis in regulating myocardial metabolism [23]. However, the mechanisms by which TRα-mediated TH signaling interacts with hypoxia and metabolic reprogramming during heart regeneration remain poorly understood. To address this gap, we investigated the role of TH signaling, particularly through TRα, in coordinating various biological processes in heart regeneration.

In this study, we focused on *thyroid hormone receptor alpha a* (*thraa*), the predominant TR isoform in the zebrafish heart, to study the role of TH signaling in heart regeneration using integrated transcriptomic, histological, and functional approaches. We employed a *thraa* mutant zebrafish model to disrupt TH signaling and conducted a time-course RNA-Seq analysis following cardiac injury [45]. Our findings revealed that reduced TH signaling enhances heart regeneration, as evidenced by reduced fibrotic scar area and improved functional recovery. Additionally, *thraa*^+/−^ mutants exhibited a transiently augmented inflammatory response and an extended CM proliferative window, suggesting that TH signaling modulates different injury responses. Notably, we observed significant alterations in the expression of HIF genes, highlighting an interplay between TH signaling and the hypoxic response. Furthermore, we identified *hif3a* as a downstream transcriptional target of *thraa*, and validation experiments using *hif3a*^+/−^ mutant zebrafish confirmed its crucial role in heart regeneration.

Collectively, our results highlight the coordination of TH signaling, hypoxia, and metabolic reprogramming during zebrafish heart regeneration. These insights enhance our understanding of the molecular mechanisms that govern the post-injury response. Furthermore, this study underscores that precise regulation and moderate reduction of TH signaling could serve as a therapeutic strategy to enhance cardiac repair in patients with myocardial injury.

## Results

### Loss of *thraa* impairs body development but enhances heart regeneration in zebrafish

**Fig. 1:**
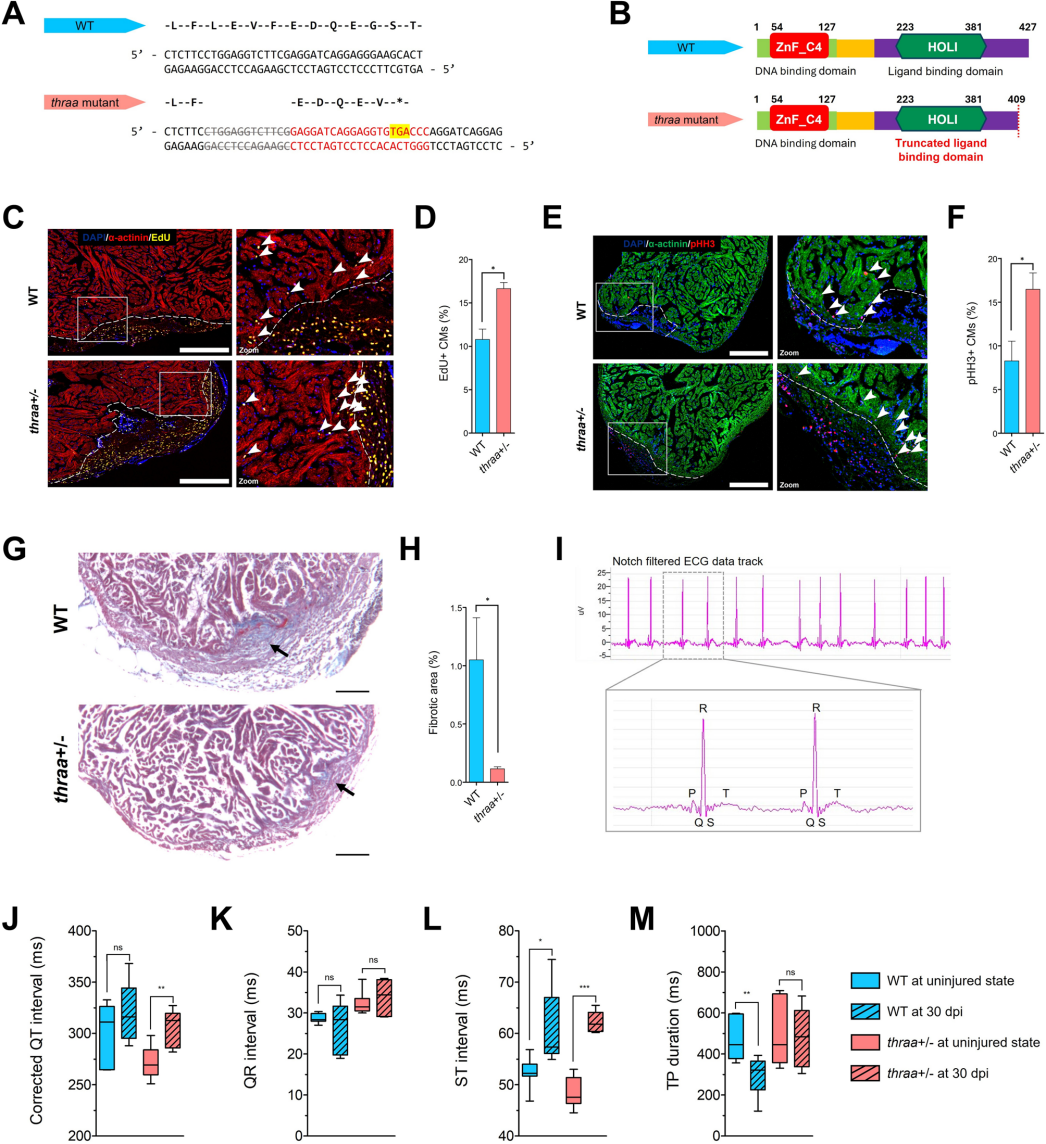
Loss of *thraa* impairs body development but enhances heart regeneration in zebrafish. (**A**) The design of *thraa* KO model in zebrafish. The amino acid (upper) and genomic (lower) sequences of both WT and *thraa* mutants. *thraa* mutants carry an early stop codon (highlighted in yellow). (**B**) Schematic diagram of the protein domain of *thraa* gene in both WT and *thraa* KO model. *thraa* mutants carry a truncated ligand-binding domain at the C-terminal. (**C**) Representative immunofluorescence images of heart sections from WT and *thraa*^*+/−*^ mutants at 7 dpi. Section images show EdU^+^ cells (yellow), α-actinin (red), and nuclei stained with DAPI (blue). Arrows in zoomed images indicate the EdU^+^ proliferating cardiomyocytes in the injured zone. Scale bar, 200 μm. (**D**) Quantification of EdU^+^ cardiomyocytes in WT and *thraa*^+/−^ mutants as shown in (**C)**. Results are shown in mean ± s.e.m; *n* = 3 in each group. (**E**) Representative immunofluorescence images of heart sections from WT and *thraa*^*+/−*^ mutants at 15 dpi. Section images show pHH3^+^ cells (red), α-actinin (green), and nuclei stained with DAPI (blue). Arrows in zoomed images indicate the pHH3^+^ proliferating cardiomyocytes in the injured zone. Scale bar, 200 μm. **(F)** Quantification of pHH3^+^ cardiomyocytes in WT and *thraa*^+/−^ mutants as shown in (**E)**. Results are shown in mean ± s.e.m; *n* = 3 in each group. **(G)** Representative images of Masson’s trichrome staining on heart sections show fibrosis of WT and *thraa*^*+/−*^ mutants at 30 dpi. Scale bar, 100 μm. (**H**) Quantification of fibrotic area in WT and *thraa*^+/−^ mutants as shown in **(G)**. Results are shown in mean ± s.e.m; *n* = 6 (WT) and 5 (*thraa*^+/−^ mutants). **(I)** Representative images of the Notch filtered zebrafish ECG signal track. P, Q, R, S, and T waves of ECG cycles are labelled in the zoomed ECG signal track. (**J-M**) Quantification of ECG signals in both WT and *thraa*^+/−^ mutants at uninjured state and 30 dpi. Quantification of corrected QT interval (**J**), QR interval (**K**), ST interval (**L**), and TP duration (**M**). *n* = 7 (uninjured WT), 7 (WT at 30 dpi), 8 (uninjured *thraa*^+/−^ mutants), and 6 (*thraa*^+/−^ mutants at 30 dpi). Box-and-whisker plots show the median with the central line, upper and lower quartiles with the edges of the box, and minimum and maximum values with whiskers. ns, *P* > 0.05; * *P* < 0.05; ** *P* < 0.01; *** *P* < 0.001 by two-tailed unpaired Student’s *t*-test (**D**, **F**, **H**) or unpaired *t*-test with Welch’s correction (**J-M**)

TH is known as a master regulator of development and metabolism, and its homeostasis is essential for maintaining normal physiological function. Clinical studies have shown that mutations in TRα or hypothyroidism can lead to growth retardation, developmental delays, coarse facial features, and constipation [46–48]. At the molecular level, TR mutations cause TH resistance by disrupting receptor-mediated transcriptional regulation. To investigate the role of TRα in zebrafish, we used a *thraa* (ENSDARG00000000151) mutant line, generated by Han *et al.*, which carries an 8-bp insertion causing a frameshift mutation in the ligand-binding domain (LBD) (Fig. 1A, B) [49]. This mutation abolishes TH binding to TRα and impairs downstream transcriptional regulation. Consistent with the TH-resistant phenotype, *thraa* knockout (KO) mutants exhibited developmental and cardiac defects [45, 49]. Our findings revealed that *thraa* mutants exhibited reduced body weight, while heart weight and the heart-to-body weight ratio showed no significant changes (Supplementary Fig. S1A-C). Notably, homozygous mutants (*thraa*^−/−^) displayed structural cardiac abnormalities, such as a thinner myocardial wall and reduced trabeculation in the ventricle (Supplementary Fig. S1D-F). To further investigate the cardiac phenotype, we performed bulk RNA-Seq on adult mutant hearts, comparing *thraa*^−/−^ mutants with WT to elucidate the transcriptomic effects of *thraa* KO (Supplementary File 1). Gene Ontology (GO) term analysis revealed altered angiogenesis, immune system development, and cardiac muscle development in *thraa*^−/−^ mutants (Supplementary Fig. S1G, Supplementary File 2). Additionally, the expression of CM maturation marker genes, such as *myh7ba*, *tcap*, *tpm3*, and *mef2ca*, was significantly reduced in *thraa*^−/−^ mutant hearts (Supplementary Fig. S1H). Notably, *thraa* itself is involved in regulating cardiac muscle cell differentiation.

To assess the role of *thraa* in heart regeneration, we first examined the regenerative response of both *thraa*^+/−^ and *thraa*^−/−^ mutants following cardiac injury. Unexpectedly, most *thraa*^−/−^ mutants died within 1 day post-injury (dpi) (survival rate: 14.3%), suggesting that complete loss of TRα-mediated TH signaling is lethal after cardiac injury (Supplementary Fig. S1I). Therefore, we focused on *thraa*^+/−^ mutants, which showed a comparable survival rate to WT (survival rates: 97.6% in WT; 100% in *thraa*^+/−^ mutants). We next evaluated whether the regenerative response was altered in *thraa*^+/−^ mutants by assessing CM proliferation, size of fibrotic scar, and functional recovery as indicators of regeneration. *thraa*^+/−^ mutants exhibited an increased number of proliferating CMs at both 7 dpi and 15 dpi, particularly in the border zone (Fig. 1C-F). Additionally, at 30 dpi, *thraa*^+/−^ mutants showed a smaller fibrotic scar area (Fig. 1G-H). Furthermore, we assessed cardiac functional recovery using electrocardiography (ECG) at 30 dpi (Fig. 1I). Parameters reflecting ventricular electrical conduction, including corrected QT interval, ST interval, and TP duration, were analyzed. Both *thraa*^+/−^ mutants and WT demonstrated prolonged QT and ST intervals after injury, suggesting incomplete recovery of ventricular depolarization and repolarization at 30 dpi (Fig. 1J-L). Notably, TP duration, which reflects the time required for ventricular relaxation, was shortened in WT but remained unchanged in *thraa*^*+/−*^ mutants compared to their respective uninjured states (Fig. 1M). Given that fibrotic scar impairs electrical conductivity and mechanical relaxation, the shortened TP duration in WT likely reflects impaired ventricular relaxation due to persistent scar, consistent with the results shown in Fig. 1G-H.

Taken together, these findings suggest that the partial loss of TH signaling via *thraa* enhances cardiac regeneration in zebrafish, as evidenced by increased CM proliferation, reduced fibrotic scar area, and improved ventricular relaxation after injury.

### Temporal transcriptomic profiles uncover cardiac injury response in zebrafish

**Fig. 2:**
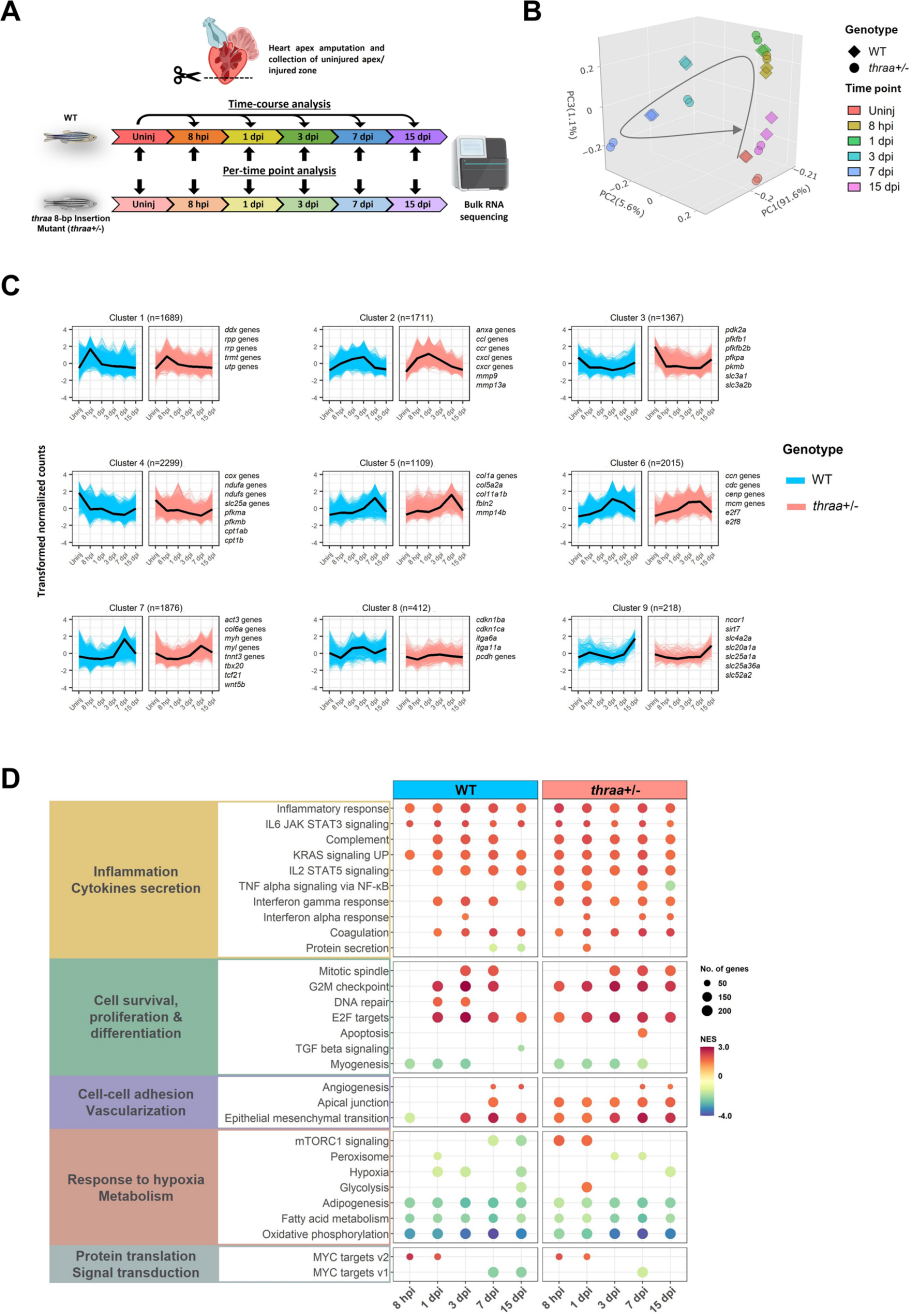
Temporal transcriptomic profiles uncover cardiac injury response in zebrafish. (**A**) Schematic diagram shows the experimental design of the time-course experiment studying heart regeneration by bulk RNA-Seq. Uninj: uninjured state; hpi: hours post injury; dpi: days post injury. Figure was created using BioRender. (**B**) 3D PCA plot of the time-course transcriptome profiles in WT and *thraa*^+/−^ mutants. Arrow illustrates the trajectory of samples from uninjured state to 15 dpi. (**C**) The hierarchical clustering of identified DEGs. DEGs were identified through time-course analysis in both WT and *thraa*^+/−^ mutants. Nine clusters were identified by the expression patterns of genes. Highlighted genes in each cluster are labelled. *n* = number of genes in cluster. (**D**) Results of GSEA on HALLMARK gene set for both WT and *thraa*^*+/−*^ through time-course analysis. NES: normalized enrichment score. +NES: upregulation; -NES: downregulation. HALLMARK gene sets are categorized according to their function and enriched genes

To elucidate the molecular mechanisms underlying the enhanced regenerative process in *thraa*^+/−^ mutants, we conducted a time-course bulk RNA-Seq experiment over a 15-day period to characterize the regenerative response and the role of TH signaling at different stages (Fig. 2A). The PCA plot was generated to visualize temporal transcriptomic changes across all samples (Fig. 2B). Notably, samples from the uninjured state and 15 dpi clustered together, suggesting that *thraa*^+/−^ mutants and WT hearts were nearly, but not fully, recovered from cardiac injury. This finding aligns with the unresolved fibrotic scar and impaired ventricular contractile function at 30 dpi in both genotypes.

We analyzed the RNA-Seq data using two strategies: time-course analysis and per-time point analysis (Fig. 2A). We first identified differentially expressed genes (DEGs) through both time-course and per-time point analyses (Supplementary File 3–5). Time-course DEG analysis revealed remarkable transcriptomic changes at 7 dpi in both genotypes compared to their respective uninjured states, highlighting 7 dpi as a critical stage in heart regeneration (Supplementary Fig. S2A, B). Surprisingly, per-time point analysis revealed that the uninjured state exhibited the most pronounced transcriptomic differences between *thraa*^+/−^ mutants and WT (Supplementary Fig. 2C, D). Consistent with the observations in *thraa*^−/−^ mutants shown in Supplementary Fig. 1G, partial loss of TH signaling in *thraa*^+/−^ mutants was sufficient to induce significant transcriptomic changes.

To further characterize regenerative response using transcriptomic profiles, DEGs from both *thraa*^+/−^ mutants and WT were clustered based on expression patterns. Using k-means clustering, nine gene clusters with distinct temporal expression patterns were identified (Fig. 2C, Supplementary File 6). Clusters 1 and 2 showed strong post-injury upregulation, encompassing genes involved in ribosomal biogenesis (e.g., ribonucleases and ribosomal RNA processing genes) and inflammatory response (e.g., chemokine ligands and receptors). Clusters 3 and 4 were consistently downregulated over time, primarily comprising metabolism-related genes, such as *pfkm*, *cpt*, *cox*, *ndufs*, and *ndufa*. Clusters 5 to 7 exhibited delayed transcriptional activation starting at 3 dpi, including genes associated with extracellular matrix (ECM) remodeling (e.g., *col5a2a*, *col11a1b*, *col1a*), cell cycle regulation (e.g., *e2f7*, *e2f8*, *ccn*, *cenp*), and cardiac muscle structure (e.g., *myh*, *myl*, *tnnt3*). Cluster 8, containing genes related to cell-cell adhesion (e.g., integrin and protocadherin genes), exhibited modest expression changes over 15 days. Cluster 9, enriched with solute carrier genes (e.g., *slc4a2a*, *slc20a1a*, *slc25a1a*), showed an exclusive upregulation at 15 dpi.

Gene set enrichment analysis (GSEA) and GO term analysis further revealed diverse injury-responsive processes in both *thraa*^+/−^ mutants and WT, including inflammatory response, cell proliferation and differentiation, vascularization, and metabolism (Fig. 2D, Supplementary Fig. S3A-D, Supplementary File 7–10). We found that inflammatory responses, cell proliferation, and metabolic alterations persisted throughout the 15-day post-injury period in both *thraa*^+/−^ mutants and WT. Notably, in *thraa*^+/−^ mutants, the mTORC1 signaling pathway and glycolysis were upregulated at early time points, with several metabolism-related genes (*hk2*, *slc2a3a*, *slc2a3b*, *fkbp2*, *g6pd*) being upregulated in the mTORC1 signaling pathway, suggesting an interplay between mTORC1 signaling and glycolysis in mediating post-injury metabolic shifts under TH signaling deficiency (Fig. 2D).

Overall, our time-series transcriptomic profiles delineate the temporal regulation of injury-responsive processes in both genotypes over a 15-day period. Particularly, the transition from 3 dpi to 7 dpi marks a shift from an inflammatory to a reparative phase, as evidenced by the activation of distinct gene sets. Moreover, this study provides insights into how zebrafish temporally coordinate multiple biological processes to achieve heart regeneration.

### Deficiency of TH signaling modulates the inflammatory response during heart regeneration

**Fig. 3:**
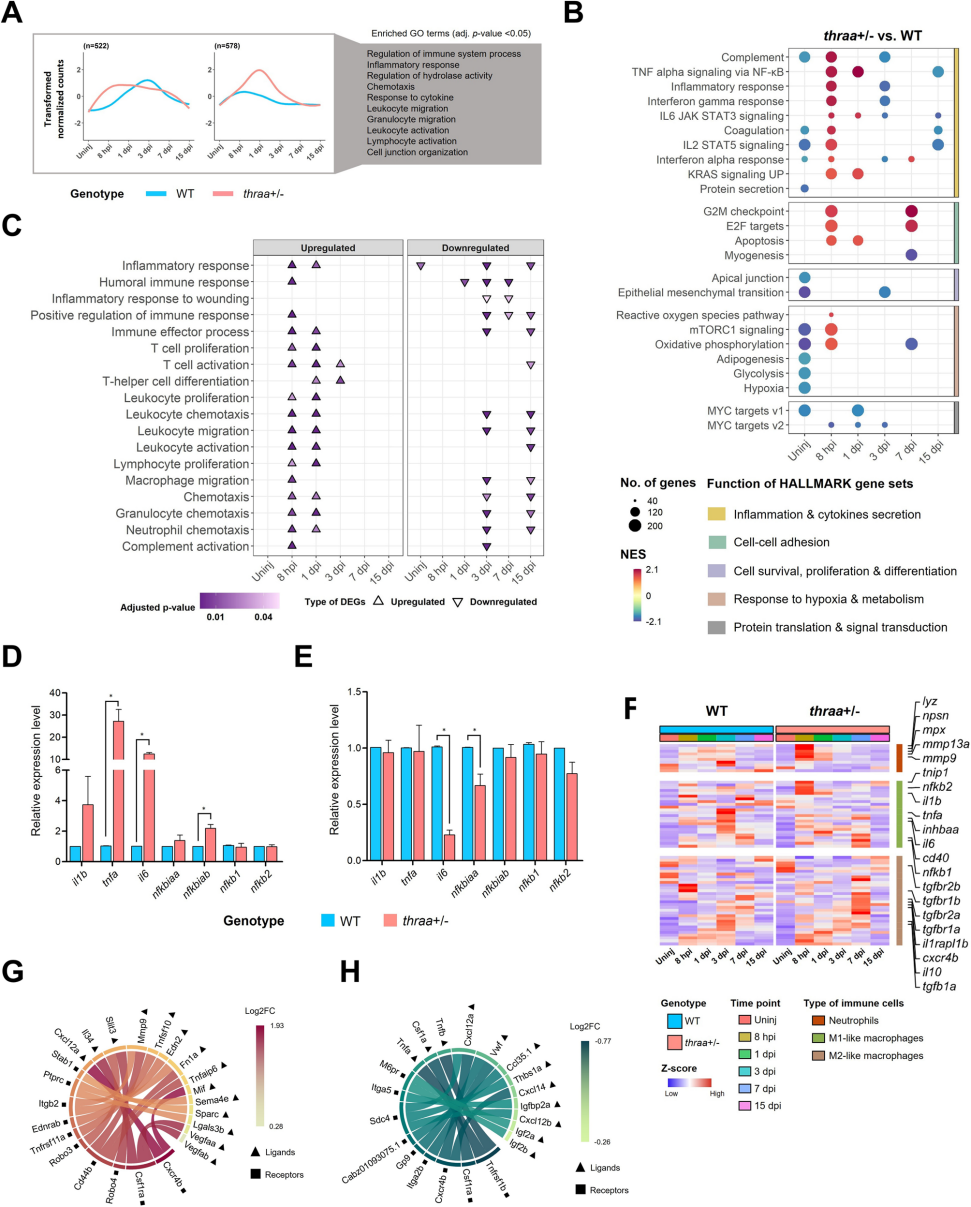
Deficiency of TH signaling modulates the inflammatory response during heart regeneration. (**A**) Line plots show the differential expression patterns of 2 gene clusters involved in inflammation. The lines in plots show the averaged expression patterns of clustered genes in both WT and *thraa*^*+/−*^ mutants. Enriched GO terms in biological process with adjusted *p*-value < 0.05 are listed along. *n* = number of genes in cluster. (**B**) Results of GSEA on HALLMARK gene set by *thraa*^*+/−*^ mutants vs. WT. NES: normalized enrichment score. +NES: upregulation in *thraa*^+/−^ mutants; -NES: downregulation in *thraa*^+/−^ mutants. (**C**) Distribution of enriched inflammation-related GO terms in upregulated and downregulated DEGs by *thraa*^+/−^ mutants vs. WT. (**D-E)** RT-qPCR analysis of selected genes in inflammation at 8 hpi (**D**) and at 15 dpi (**E**). *n* = 2 for both WT and *thraa*^+/−^ mutants at 8 hpi; *n* = 3 for both WT and *thraa*^+/−^ mutants at 15 dpi. Results are shown in mean ± s.e.m; * *P* < 0.05. Statistical significance was calculated by two-tailed unpaired Student’s *t*-test. **(F)** Heatmap shows the expression of marker genes indicating the recruitment of neutrophils and macrophages. (**G-H)** Ligand-receptor interaction analysis of the upregulated genes at 8 hpi (**G**) and downregulated genes at 15 dpi (**H**). Circle plots show the top ranked ligand-receptor pairs connected with ribbon. The color of circular ring represents the log_2_FC of genes by *thraa*^+/−^ mutants vs. WT

To precisely understand the role of TH signaling via *thraa* in zebrafish heart regeneration, we conducted a per-time point analysis along with splines clustering [50]. Notably, *thraa*^+/−^ mutants exhibited a distinct inflammatory response compared to WT, as evidenced by clusters of various inflammation-related genes (Fig. 3A, Supplementary File 11). The differential expression of genes involved in inflammation and immune cell recruitment suggests an augmented and earlier peak of the inflammatory response in *thraa*^+/−^ mutants. GSEA and GO term analysis further revealed a profound inflammatory response in *thraa*^+/−^ mutants, particularly at 8 h post-injury (hpi), while this response was attenuated during the reparative phase starting from 3 dpi (Fig. 3B, C, Supplementary Fig. S4A, B and Supplementary File 12, 13). Among the upregulated pathways, TNF-α and IL-6 signaling showed the most rapid and pronounced activation (Fig. 3B and S5A, B). To confirm the augmented inflammatory response in *thraa*^+/−^ mutants, we performed RT-qPCR analysis on both *thraa*^+/−^ mutants and WT at 8 hpi and 15 dpi (Fig. 3D, E). At 8 hpi, expression levels of inflammation-related genes, such as *tnfa*, *il6*, and *nfkbiab*, were elevated in *thraa*^+/−^ mutants, while these genes were downregulated at 15 dpi, indicating a relatively mild inflammatory response in *thraa*^+/−^ mutants during the late reparative phase.

We also observed enhanced cell cycle activity (G2M and E2F pathways) in *thraa*^+/−^ mutants at 8 hpi (Fig. 3B). This implies that activated and recruited immune cells undergo active proliferation at the injury site during the early inflammatory phase and thus contribute to the stronger inflammatory response in *thraa*^+/−^ mutants. In addition to the proliferative activity of recruited immune cells, their pro- and anti-inflammatory phenotypes also shape the inflammatory outcomes. To investigate this, we inferred immune cell composition at the injured site using a panel of marker genes (Fig. 3F) [51–53]. Neutrophils and macrophages, as primary responders in inflammation, can switch their transcriptomic signatures to adopt either pro- or anti-inflammatory properties through polarization. In *thraa*^+/−^ mutants, we observed elevated expression of pro-inflammatory neutrophil markers (e.g., *lyz*, *mmp9*, *mp13a*) and M1-like macrophage markers (e.g., *tnip1*, *tnfa*, *il1b*, *il6*) at 8 hpi. As regeneration progresses, M1-like macrophages transition to M2-like phenotype with anti-inflammatory properties, facilitating inflammation resolution. Notably, marker genes of M2-like macrophages (e.g., *tgfb1a*, *tgfbr1a/1b*, *tgfbr2a/2b*, *il10*) were significantly upregulated in *thraa*^+/−^ mutants at 7 dpi, indicating that inflammation started to resolve. These findings revealed the differences in immune cell populations and dynamic shifts in their polarization states, contributing to the transiently amplified inflammatory response in *thraa*^+/−^ mutants.

Ligand-receptor interaction analysis further revealed distinct inflammatory modulation between *thraa*^+/−^ mutants and WT (Fig. 3G, H). Several ligand-receptor pairs with inflammation-modulating functions, including those in the Tumor Necrosis Factor (TNF) family, were upregulated at 8 hpi but downregulated at 15 dpi. Of note, the *cxcl12a*-*cxcr4b* chemokine signaling pair, found at both 8 hpi and 15 dpi, is known to regulate inflammation in AMI by promoting immune cell infiltration and enhancing TNFα expression [54, 55]. These findings highlight the role of ligand-receptor interactions in contributing to the altered inflammatory response in *thraa*^+/−^ mutants during both inflammatory and reparative phases.

Collectively, these findings demonstrate that *thraa*^+/−^ mutants, characterized by reduced TH signaling, exhibit an earlier and amplified inflammatory response following cardiac injury. The post-injury inflammatory response was significantly augmented, as evidenced by strong upregulation of inflammation-modulatory pathways and altered transcriptomic signatures of immune cells. However, the enhanced inflammatory response was transient and decreased during the late reparative phase.

### Attenuation of TH signaling enhances heart regeneration by extending the time window for cardiomyocyte proliferation

**Fig. 4:**
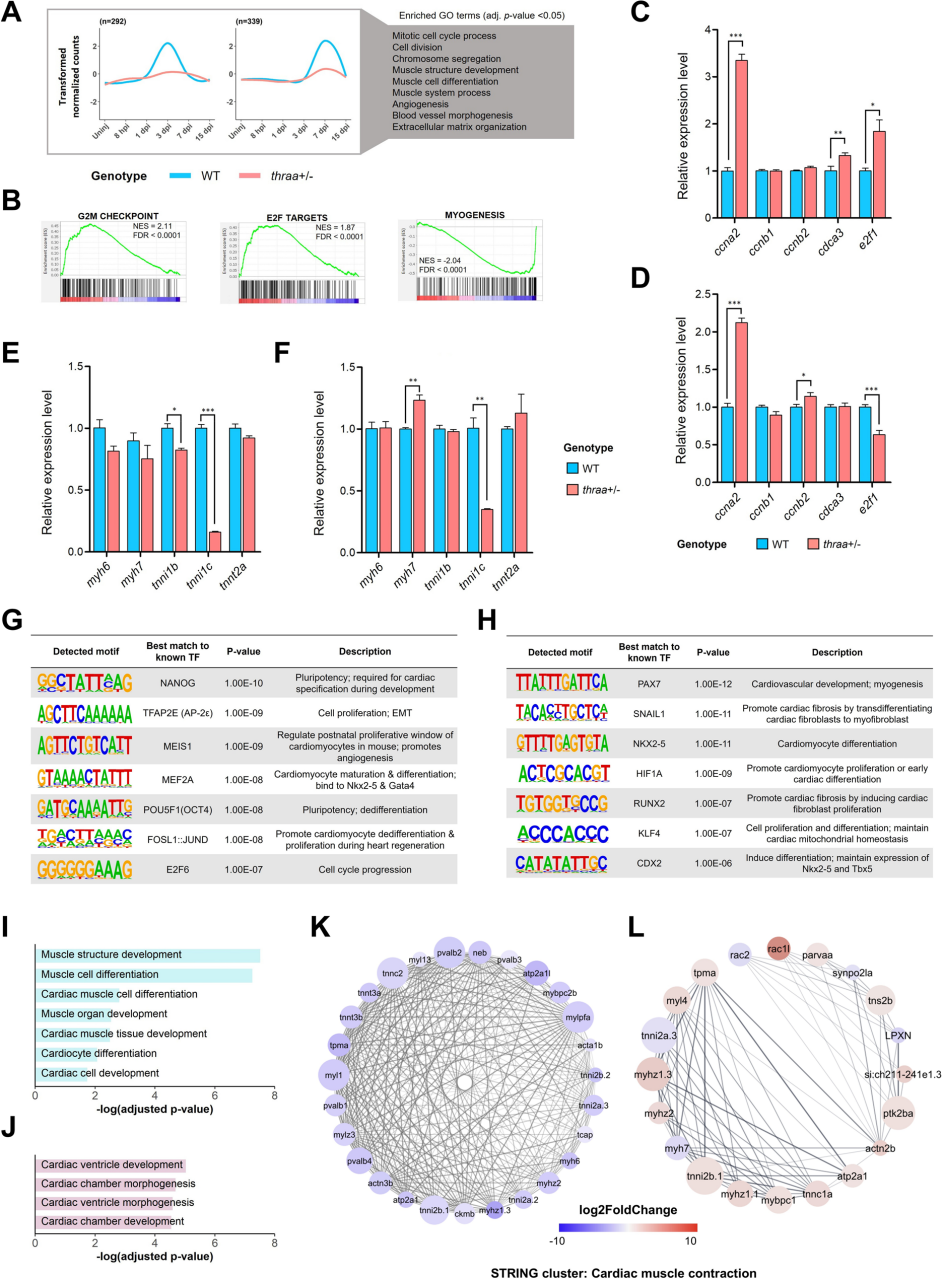
Attenuation of TH signaling enhances heart regeneration by extending the time window for cardiomyocyte proliferation. (**A**) Line plots show the differential expression patterns of 2 gene clusters involved in regenerative processes. The lines in plots show the averaged expression patterns of clustered genes in both WT and *thraa*^*+/−*^ mutants. Enriched GO terms in biological process with adjusted *p*-value < 0.05 are listed along. *n* = number of genes in cluster. (**B**) Selected enrichment plots of GSEA as shown in Fig. 3B by *thraa*^+/−^ mutants vs. WT at 7 dpi. (**C-D**) RT-qPCR analysis of selected genes in cell cycle regulation at 3 dpi **(C**) and at 7 dpi (**D**). *n* = 6 (WT at 3 dpi), 9 (*thraa*^+/−^ mutants at 3 dpi), 6 (WT at 7 dpi) and 5 (*thraa*^+/−^ mutants at 7 dpi). (**E-F**) RT-qPCR analysis of selected genes in cardiomyocyte differentiation at 7 dpi (**E**) and at 15 dpi (**F**). *n* = 3 for both WT and *thraa*^+/−^ mutants at 7 dpi and 15 dpi. In (**C-F**), results are shown in mean ± s.e.m; * *P* < 0.05; ** *P* < 0.01; *** *P* < 0.001. Statistical significance was calculated by two-tailed unpaired Student’s *t*-test. (**G-H**) Motif enrichment analysis of upregulated (**G**) and downregulated (**H**) DEGs at 7 dpi by *thraa*^+/−^ mutants vs. WT. The function of best matched transcription factors is described in table. (**I-J**) Selected enriched GO terms of downregulated DEGs at 7 dpi (**I**) and upregulated DEGs at 15 dpi (**J**) by *thraa*^+/−^ mutants vs. WT. (**K-L**) PPI network of STRING cluster with cardiac muscle contraction function. PPI network of downregulated DEGs at 7 dpi (**K**) and upregulated DEGs at 15 dpi (**L**) by *thraa*^+/−^ mutants vs. WT. The node color represents the log_2_FC of genes

In addition to the inflammatory response, replacing dead or damaged cardiac tissue is another crucial event of heart regeneration. Successful regeneration involves replacing fibrotic tissue with newly formed and functional cardiac muscle, thereby restoring cardiac function. In addition to the gene clusters discussed previously, two additional gene clusters, identified through the splines clustering method, exhibited distinct responses in cell proliferation and muscle cell differentiation between *thraa*^+/−^ mutants and WT (Fig. 4A, Supplementary File 11).

As shown in Fig. 1C-F, *thraa*^+/−^ mutants exhibited enhanced CM proliferation at both 7 dpi and 15 dpi compared to WT. Consistently, GSEA analysis revealed that CM proliferation was favored over muscle cell differentiation in *thraa*^+/−^ mutants at 7 dpi (Figs. 3B and 4B, Supplementary File 12). Notably, CM proliferation peaked at 3 dpi in WT, whereas in *thraa*^+/−^ mutants, the proliferative window was extended to 7 dpi, suggesting that more CMs proliferate in response to injury (Supplementary Fig. S6A). As expected, in WT, CM differentiation (myogenesis) peaked at 7 dpi following the proliferation peak, indicating that newly proliferated cells differentiate into functional CMs for regeneration (Supplementary Fig. S6B). In contrast, *thraa*^+/−^ mutants showed no clear CM differentiation peak, with only a few myogenic genes upregulated at 15 dpi (Supplementary Fig. S6B). This suggests that CM differentiation in *thraa*^+/−^ mutants may occur after 7 dpi due to the prolonged proliferative window and inverse relationship between proliferation and differentiation [56]. To validate these findings, we examined the expression of marker genes for cell proliferation and CM differentiation at various time points (Fig. 4C-F). As expected, positive cell cycle regulators were upregulated in *thraa*^+/−^ mutants at 3 and 7 dpi, with *ccna2* showing the strongest induction (Fig. 4C, D). Additionally, all CM differentiation markers showed downregulation in *thraa*^+/−^ mutants at 7 dpi, with *tnni1c* showing consistent downregulation until 15 dpi (Fig. 4E, F). However, one of the examined differentiation markers, *myh7*, was significantly upregulated at 15 dpi.

Transcription factor (TF) analysis further revealed the upstream regulators contributing to the altered regenerative response at 7 dpi (Fig. 4G, H). Specifically, an imbalanced transcriptional program between proliferation and differentiation was observed in *thraa*^+/−^ mutants. Upregulated genes were regulated by TFs modulating CM proliferation (e.g., AP-2*ε*, MEIS1, FOSL1::JUND, E2F6) and pluripotency (e.g., NANOG, OCT4), while downregulated genes were regulated by TFs associated with cardiovascular development, CM differentiation, and cell identity (e.g., PAX7, HIF1A, NKX2.5, KLF4, CDX2). This implies that upstream TFs in *thraa*^+/−^ mutants favor CM proliferation over differentiation at 7 dpi.

Interestingly, despite the extended CM proliferative window and delayed CM differentiation in *thraa*^+/−^ mutants at 7 dpi, we observed enhanced cardiac tissue regeneration at 15 dpi. In line with the GSEA analysis, GO term analysis revealed that muscle cell differentiation and cardiac cell development were negatively enriched in *thraa*^+/−^ mutants at 7 dpi (Fig. 4I, Supplementary File 13). However, processes related to cardiac chamber and ventricular development became positively enriched at 15 dpi (Fig. 4J, Supplementary File 13). Additionally, STRING-based protein-protein interaction (PPI) networks further support this notion, as evidenced by an opposing expression trend of cardiac muscle contraction genes between 7 and 15 dpi in *thraa*^+/−^ mutants (Fig. 4K, L). At 15 dpi, cardiac muscle contraction genes exhibited higher expression levels in *thraa*^+/−^ mutants compared to WT. These results indicate that *thraa*^+/−^ mutants achieve enhanced replacement of damaged cardiac tissue and improved functional recovery by 15 dpi.

In summary, these findings demonstrate that *thraa*^+/−^ mutants exhibit an altered regenerative response during the late reparative phase, particularly during 7 dpi to 15 dpi, characterized by an extended proliferative window in CMs and enhanced cardiac ventricular development and contraction. These changes collectively suggest that reduced TH signaling enhances cardiac tissue replenishment and contributes to improved regenerative outcomes by 15 dpi.

### Heart regeneration is characterized by diverse and dynamic metabolic reprogramming

Myocardial metabolic reprogramming is recognized as a protective adaptation following ischemic injury, shifting from aerobic to anaerobic pathways under the hypoxic condition in ischemic myocardium. Additionally, it has been previously reported that TH primarily regulates metabolism, contributing to differential injury responses and regenerative outcomes [23]. However, a comprehensive understanding of various metabolic pathways that respond to cardiac injury remains elusive. Therefore, in this study, we elucidated how reduced TH signaling influences these pathways in response to cardiac injury and further delineated the dynamic and stage-specific changes in diverse metabolic pathways.

The per-time point GSEA analysis revealed that OXPHOS activity is altered in *thraa*^+/−^ mutants at 8 hpi and 7 dpi, correlating with the peak of transiently intensified inflammatory response and the extended CM proliferative window (Fig. 3B, Supplementary File 12). Compared to WT, *thraa*^+/−^ mutants exhibited a higher OXPHOS activity during the early inflammatory phase, while its activity was relatively lower during the reparative phase. This suggests that reduced TH signaling exerts differential regulation across these phases of cardiac regeneration.

**Fig. 5:**
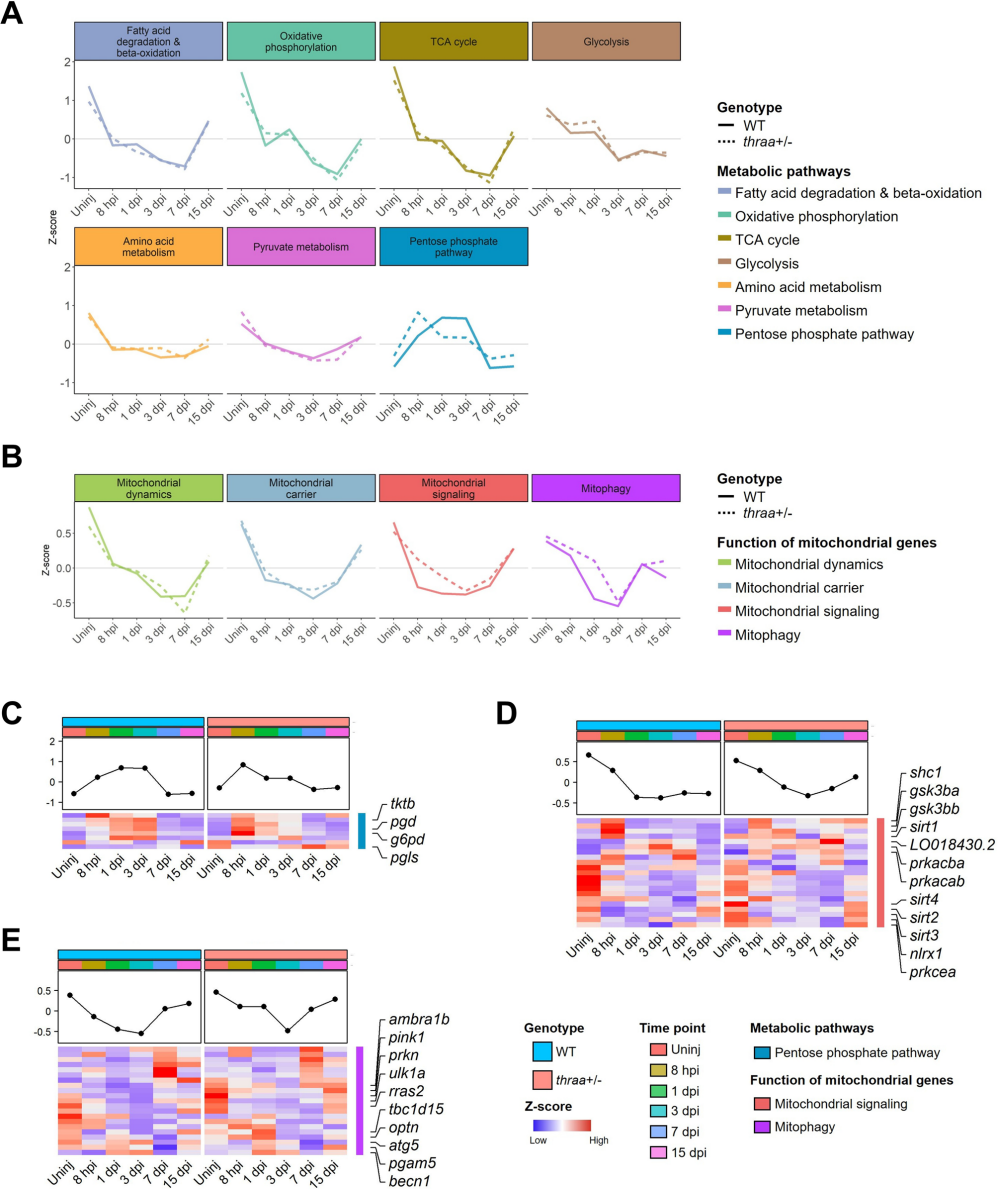
Heart regeneration is characterized by diverse and dynamic metabolic reprogramming. (**A-B**) The line plots show the averaged expression patterns of genes categorized by metabolic pathways (**A**) and mitochondrial function (**B**) in both WT and *thraa*^+/−^ mutants. (**C-E**) Heatmaps show the expression of genes in pentose phosphate pathway (**C**), mitochondrial signaling (**D**), and mitophagy (**E**) as shown in Fig. 5A and B

Next, we explored the temporal regulation of various metabolic pathways to further investigate the metabolic shifts and the changes in mitochondrial function by mapping our time-course transcriptomic data to the mitoXplorer 2.0 database (Fig. 5A, B, Supplementary File 14) [57]. Fatty acid oxidation (FAO), along with the closely linked OXPHOS and TCA cycle, predominated in the uninjured state, but their activity sharply declined following cardiac injury, underscoring their central role in myocardial metabolism and sensitivity to injury (Supplementary Fig. S7A-C). Similarly, glycolytic genes exhibited a comparable downward expression trend (Supplementary Fig. S7D). Notably, these metabolic pathways exhibited a plateau between 8 hpi and 1 dpi, followed by a further reduction. Oxidative pathways, including FAO, OXPHOS, and the TCA cycle, reached their lowest activity at 7 dpi. The plateau phase and the timing of the lowest activity level align with the peak of the inflammatory response and cardiac tissue regeneration, respectively, suggesting that these injury responses occur within a distinct metabolic microenvironment. Until 15 dpi, we observed a partial restoration of myocardial energy metabolism through the reactivation of these oxidative metabolic pathways. Surprisingly, glycolysis exhibited its lowest activity at 3 dpi and showed no significant gene upregulation at later time points, underscoring its distinct regulatory dynamics in myocardial metabolism, particularly during the late reparative phase.

Additionally, amino acid and pyruvate metabolism are two other pathways that may contribute to myocardial energy metabolism by fueling metabolites, such as acetyl-CoA and α-ketoglutarate, to the TCA cycle for ATP production (Supplementary Fig. S7E, F), indicating their roles in supporting basal metabolism in the heart. However, they are less prominent than oxidative metabolic pathways, as only about half of their genes show high expression at homeostasis. Similarly, they exhibited reduced metabolic activity in response to cardiac injury, with no restoration of activity observed until 15 dpi.

Surprisingly, the pentose phosphate pathway (PPP) was the only pathway strongly upregulated upon cardiac injury, particularly through the increased expression of *tktb*, *pgd*, and *g6pd* (Fig. 5C). Moreover, the peak in differential expression of PPP between *thraa*^+/−^ mutants and WT resembles that observed in the inflammatory response, as shown in Fig. 3A, suggesting an interplay between PPP and inflammatory modulation mediated by TH signaling [58].

Apart from energy production, mitochondria also shape the local microenvironment after injury by releasing damage-associated molecular patterns (DAMPs) during apoptosis, thereby modulating inflammation and CM survival and proliferation [24, 59–61]. We therefore checked the expression level of genes related to mitochondrial function and identified disrupted mitochondrial activities in *thraa*^+/−^ mutants (Fig. 5B). Genes involved in mitochondrial dynamics, transport, and signaling were significantly downregulated in *thraa*^+/−^ mutants after cardiac injury (Fig. 5D, Supplementary Fig. S7G, H). The sharp decline in these genes mirrors that observed in oxidative metabolic pathways, suggesting the central regulatory role of mitochondria in these processes. In mitochondrial signaling, *thraa*^+/−^ mutants exhibited reduced expression of *gsk3ba* and *gsk3bb* at 8 hpi, inhibition of which is known to improve myocardial dysfunction (Fig. 5D) [62]. Moreover, particularly in *thraa*^+/−^ mutants, we noticed that a group of sirtuin genes (i.e., *sirt1*/*2*/*3*/*4*), known for their cardioprotective functions, were highly expressed at 15 dpi [63]. Mitophagy, a process that maintains mitochondrial homeostasis, has been shown to promote heart regeneration in zebrafish by inducing CM proliferation, and modulating collagen deposition and resolution [64]. Our data further revealed that several mitophagy-related genes (e.g., *tbc1d15*, *atg5*, *pgam5*,* becn1*) were highly expressed in *thraa*^+/−^ mutants at 1 dpi, indicating the role of mitophagy in regulating the post-injury inflammatory microenvironment (Fig. 5E) [65].

Taken together, our findings demonstrate that TH signaling differentially regulates metabolic pathways, particularly OXPHOS and PPP, across inflammatory and reparative phases, while also governing mitochondrial turnover and function, thereby enhancing heart regeneration in *thraa*^+/−^ mutants.

### Disruption of *thraa* affects hypoxic response and its target *hif3a* is required for heart regeneration

Hypoxia can reprogram cellular metabolism in the heart following ischemic injury, reducing oxygen consumption, reactive oxygen species (ROS) production, and the resulting oxidative stress within the ischemic tissue microenvironment, leading to a beneficial effect in heart regeneration in both mice and zebrafish [66, 67]. HIF1α, an identified TH-dependent gene, plays a role in the transcriptional regulation of mitochondrial metabolism and cell cycle progression in the heart [68–71]. In the context of the uninjured adult zebrafish heart, our data showed that complete KO of *thraa* significantly reduced the expression of *hif1aa*, while increasing the expression of *hif2ab* and *hif3a* (Supplementary Fig. S8A). These findings suggest that TH signaling via *thraa* regulates the transcription of all three HIFα family members in the zebrafish heart.

**Fig. 6:**
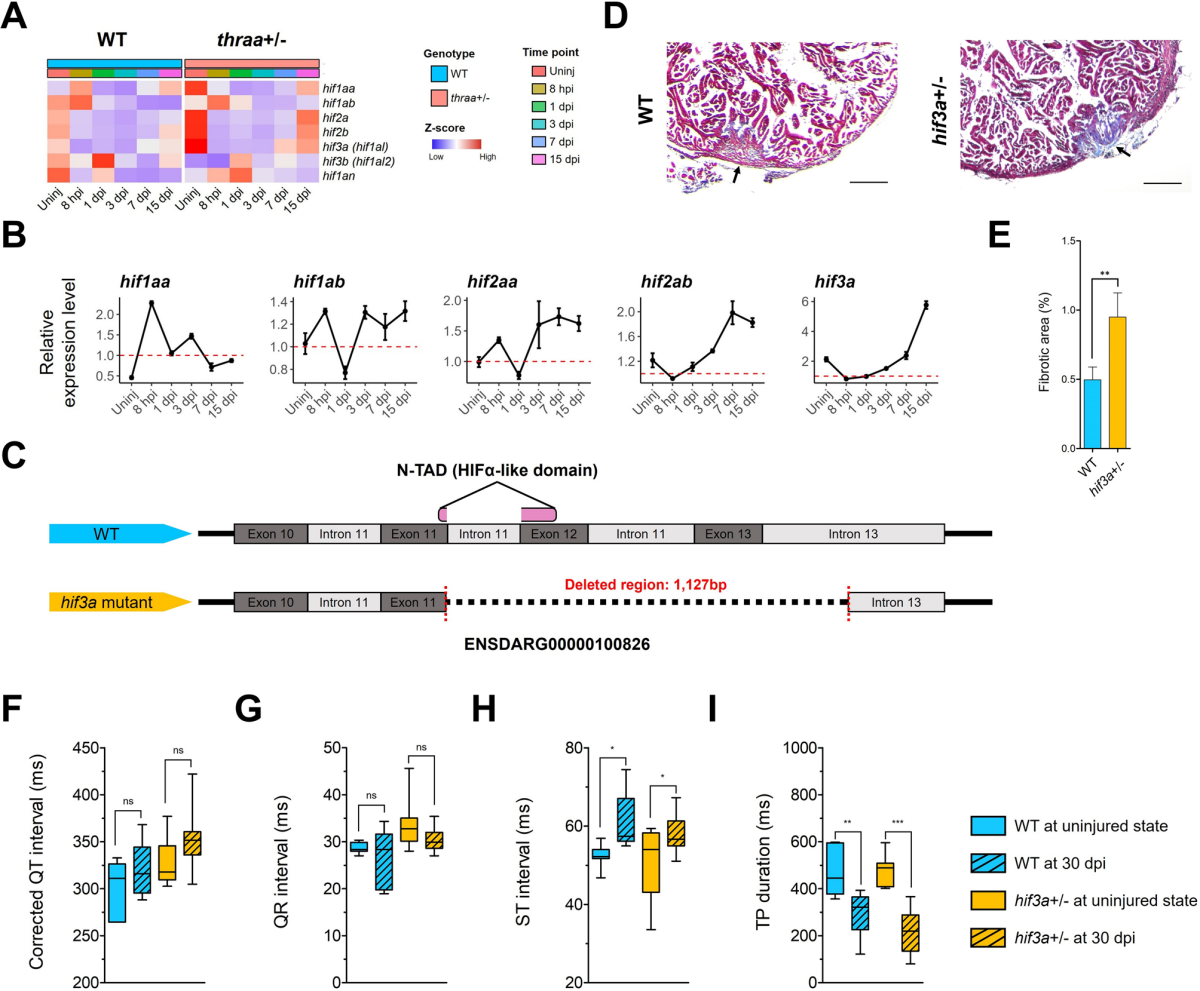
Disruption of *thraa* affects hypoxic response and its target *hif3a* is required for heart regeneration. (**A**) Heatmap shows the expression of hypoxia genes in transcriptomic profiles in both WT and *thraa*^+/−^ mutants. (**B**) RT-qPCR analysis of selected hypoxia genes. The red dashed line indicates the normalized relative expression level of WT at 1. Results are shown in mean ± s.e.m; *n* = 3 at each time point for both WT and *thraa*^+/−^ mutants. (**C**) The design of *hif3a* KO model in zebrafish. >1 kb of genomic sequences was deleted by CRISPR/Cas9 spanning from exon 11 to intron 13. The deleted region covers the region translating N-TAD (HIFα-like domain) in Hif3α protein. (**D**) Representative images of Masson’s trichrome staining on heart sections show fibrosis of WT and *hif3a*^*+/−*^ mutants at 30 dpi. Scale bar, 100 μm. (**E**) Quantification of fibrotic area in WT and *hif3a*^+/−^ mutants as shown in (**D**). Results are shown in mean ± s.e.m; *n* = 7 for WT and *n* = 7 for *hif3a*^+/−^ mutants. (**F-I**) Quantification of ECG signals in both WT and *hif3a*^+/−^ mutants at the uninjured state and 30 dpi. corrected QT interval (**F**), QR interval (**G**), ST interval (**H**) and TP duration (**I**). *n* = 7 (uninjured WT), 7 (WT at 30 dpi), 10 (uninjured *hif3a*^+/−^ mutants), and 6 (*hif3a*^+/−^ mutants at 30 dpi). Box-and-whisker plots show the median with the central line, upper and lower quartiles with the edges of box, and minimum and maximum values with whiskers. ns, *P* > 0.05; * *P* < 0.05; ** *P* < 0.01; *** *P* < 0.001 by two-tailed unpaired Student’s *t*-test (**E**) or unpaired *t*-test with Welch’s test (**F-I**)

We next hypothesized that the differential transcriptional response of HIFs could also be observed in *thraa*^+/−^ mutants following cardiac injury. As expected, both transcriptomic profiles and RT-qPCR analysis revealed a disrupted hypoxic response in *thraa*^+/−^ mutants, characterized by altered expression patterns of all HIF genes (Fig. 6A, B). Notably, we observed a 6-fold increase in *hif3a* expression at 15 dpi (Fig. 6B). Furthermore, our RT-qPCR data demonstrated that *hif3a* expression is regulated by *thraa* in a dosage-dependent manner (Supplementary Fig. S8B). To support this, we identified hormonal regulation and binding of TRα to the Hif3a promoter in mouse hearts, indicating that TRα binding suppresses Hif3a expression (Supplementary Fig. S8C) [23, 72]. Together, these findings support our hypothesis that *thraa* directly regulates *hif3a* transcription, contributing to the heart regenerative response in zebrafish.

HIF3α is thought to be a negative regulator of both HIF1α and HIF2α actions, either by triggering a unique transcriptional program or in a dominant-negative manner [44]. Unlike HIF1α and HIF2α, HIF3α contains only a single transactivation domain at the N-terminus (N-TAD) and lacks a C-terminal transactivation domain (C-TAD). These distinctive domain features are conserved across species (Supplementary Fig. S8D). Therefore, we hypothesized that elevated HIF3α expression may aid in resolving the hypoxic response by inhibiting or modulating the transcriptional activation of hypoxia-response elements (HREs). To test this hypothesis, we generated *hif3a* (ENSDARG00000100826) KO mutant zebrafish to investigate its role in heart regeneration (Fig. 6C). The *hif3a* mutants carry a > 1 kb deletion in the genomic region encoding the sole HIFα-like transactivation domain (N-TAD), resulting in disrupted transcript splicing of *hif3a* (Supplementary Fig. S8E, F). In this mutant line, we observed a clear phenotypic difference between *hif3a*^−/−^ mutants and WT, with *hif3a*^−/−^ mutants exhibiting a significantly enlarged atrium compared to WT (Supplementary Fig. S8G-I).

We evaluated the role of *hif3a* in heart regeneration by examining fibrotic scar area in *hif3a*^+/−^ mutants at 30 dpi. We observed a larger fibrotic scar area in *hif3a*^+/−^ mutants compared to WT (Fig. 6D, E), suggesting that *hif3a* expression, particularly during the late reparative phase, is essential for effective heart regeneration. To assess the functional recovery, we performed ECG analysis at 30 dpi in both *hif3a*^+/−^ mutants and WT (Fig. 6F-I). Consistent with observations in *thraa*^+/−^ mutants, *hif3a*^+/−^ mutants were not fully recovered from cardiac injury, as evidenced by altered ventricular conduction parameters. Additionally, the ECG revealed a greater reduction in TP duration in *hif3a*^+/−^ mutants compared to WT, suggesting more impaired ventricular relaxation due to persistent scarring.

Together, these findings underscore the critical role of *hif3a* in zebrafish heart regeneration and its functional link with *thraa*. Upregulation of *hif3a* appears to be necessary for orchestrating the regenerative response in zebrafish.

## Discussion

**Fig. 7:**
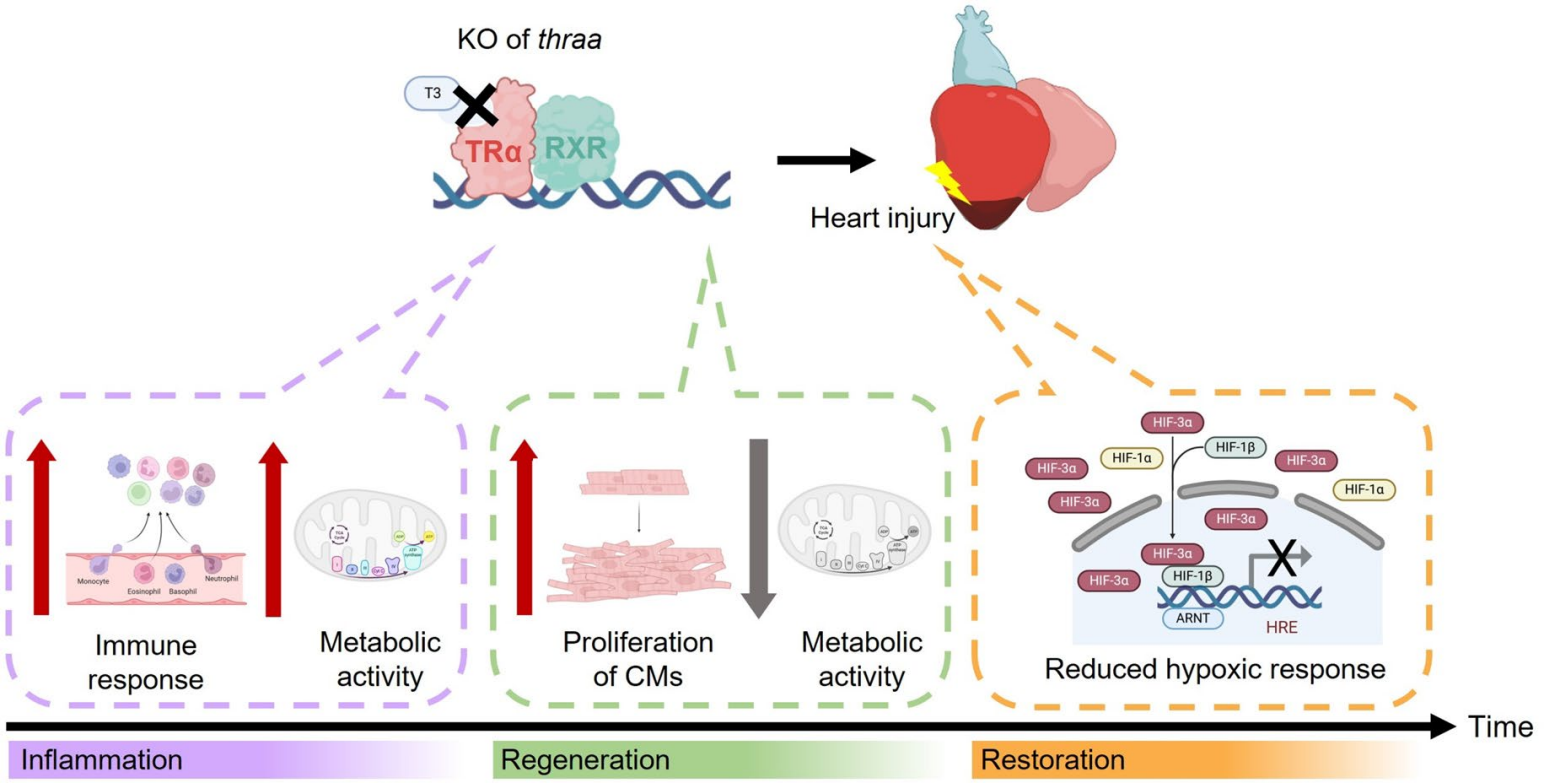
Schematic diagram illustrating the effect of TH signaling during heart regeneration in zebrafish. Attenuated TH signaling shows a beneficial effect on heart regeneration by enhancing the inflammatory response in the early phase, promoting cardiomyocyte proliferation in the late reparative phase, and modulating the hypoxic response via HIF3α. TH signaling plays a role in regulating metabolic activity, coinciding with the transiently intensified inflammation and the enhanced cardiomyocyte regenerative response. Moreover, TH signaling via *thraa* regulates the hypoxic response by modulating the expression of *hif3a* through direct transcriptional regulation. The figure was created using BioRender

Heart regeneration, a biological process that reverses myocardial damage caused by acute myocardial infarction (AMI), fails to happen in adult humans. As a result, AMI often leads to heart failure. Moreover, regenerative potential varies significantly across species and developmental stages [73]. In 2019, Hirose *et al.* introduced a novel concept to elucidate the role of the TH/TRα signaling axis in basal metabolism and its correlation with heart regenerative outcomes [23]. Their study demonstrated that adult mice with CM-specific inactivation of TRα exhibit reduced basal oxidative metabolism (i.e., OXPHOS and TCA cycle) while retaining regenerative potential through promoting CM proliferation and fibrotic scar resolution. These data suggest that attenuation of TH/TRα signaling has a beneficial effect on heart regeneration in mice. In addition to the findings of Hirose *et al.*, our study extended the understanding of the TH/TRα signaling axis and further elaborated its underlying molecular mechanisms in response to cardiac injury. In our study, we employed *thraa* and *hif3a* mutant zebrafish and performed a time-course experiment to elucidate how TH/TRα signaling orchestrates the inflammatory response and cardiac tissue regeneration via metabolic regulation, as well as the interaction between TH/TRα signaling and hypoxia via HIF3α (Fig. 7).

We first observed an altered temporal regulation of inflammatory response in *thraa*^+/−^ mutants, which exhibited a transiently augmented response upon cardiac injury (Fig. 3). As the initial immune response serves as the first line of defense against cardiac injury, and its precise activation and programmed resolution are critical for downstream regenerative events, such as ECM remodeling for fibrosis and neovascularization [74]. Previous studies have emphasized that inflammation acts as a double-edged sword: excessive leukocyte infiltration can impair regeneration, while insufficient inflammation may disrupt fibrosis and dampen the regenerative response [75, 76]. In our *thraa*^+/−^ mutants, the peak of inflammation was shifted to earlier time points, resulting in a relatively mild inflammatory response during the late reparative phase. Notably, we observed significant alterations in inflammatory response at 8 hpi and 15 dpi, as evidenced by the activation of inflammatory signaling pathways, recruitment and phenotypic switches of immune cells, and interactions between inflammation-modulating ligands and receptors.

Replacing dead myocardial tissue with newly formed CMs is essential for restoring physiological function after cardiac injury. CMs in zebrafish can undergo dedifferentiation and cell cycle re-entry during heart regeneration [77, 78]. Consistent with the findings of Hirose *et al.*, who demonstrated impaired CM proliferation in zebrafish treated with exogenous T3, our *thraa*^+/−^ mutants exhibited enhanced CM proliferation during heart regeneration, suggesting that CM proliferation is modulated by TH through TRα. To further elucidate the dynamics of this regenerative process, we conducted a time-course analysis that uncovered two distinct waves of cell proliferation. The first wave, observed at early time points, coincides with immune cell recruitment and activation, while the second wave may reflect CM proliferation, as evidenced by IF staining and the subsequent peak of cardiac muscle differentiation at 7 dpi. Importantly, we observed an extended time window for CM proliferation in *thraa*^+/−^ mutants, spanning from 3 dpi to 7 dpi, while WT showed a peak only at 3 dpi (Fig. 4). Given the inverse relationship between proliferation and differentiation, CM differentiation was only evident in WT at 7 dpi, while *thraa*^+/−^ mutants showed sustained CM proliferation [56]. This extended proliferative window in *thraa*^+/−^ mutants suggests that more newly formed CMs are present in the injured zone, contributing to improved regenerative outcomes [79]. In line with this notion, our ECG data demonstrated improved cardiac functional recovery as evidenced by enhanced ventricular contraction, further suggesting that attenuated *thraa*-mediated TH signaling enhances zebrafish heart regeneration.

Furthermore, we observed that differential metabolic activity coincides with the peaks during both inflammatory and reparative phases, suggesting an interplay between TH-mediated metabolism and the injury response (Fig. 5). During the inflammatory phase, particularly at 8 hpi, *thraa*^+/−^ mutants exhibited increased OXPHOS and PPP activity, which was associated with an enhanced inflammatory response. During the inflammatory phase, various immune cells are recruited to the injured site, exhibiting a flexible metabolic switch to meet the energy demands of their defensive actions. Specifically, chemokine-attracted neutrophils primarily rely on mitochondrial OXPHOS, whereas M1-like macrophages switch from OXPHOS to glycolysis and PPP [58, 80–82]. Therefore, the distinct metabolic states between *thraa*^+/−^ mutants and WT may imply the variations in immune cell composition at the injured site. As regeneration progresses to the reparative phase, WT and *thraa*^+/−^ mutants exhibited differential OXPHOS activity at 7 dpi, with *thraa*^+/−^ mutants displaying an extended CM proliferative window. The level of OXPHOS activity may reflect the maturation state of CMs, as terminally differentiated CMs predominantly rely on OXPHOS and FAO for energy production. However, in *thraa*^+/−^ mutants, we did not observe significant upregulation of glycolysis compared to that in WT, which is known to be the primary metabolic pathway supporting CM proliferation [35].

As our study in *thraa*^+/−^ mutants utilized bulk RNA-Seq to delineate the injury response within the microenvironment of the injured zone, it remains to be investigated how TH-mediated metabolism modulates this response in individual cell types, leading to enhanced regenerative outcomes. For instance, further investigations are required to clarify the role of TH/TRα signaling in modulating the phenotypic switch of various immune cell types. Moreover, cardiac regeneration involves the orchestration of multiple processes to achieve complete regeneration. In this study, however, we observed no significant alterations in other critical processes between *thraa*^+/−^ mutants and WT, such as angiogenesis and ECM degradation, which are known to be modulated by TH [83–85]. Additionally, in this study, we hypothesized that TH-mediated metabolism primarily drives the altered regenerative response in *thraa*^+/−^ mutants. However, we cannot exclude the possibility that TRα directly regulates genes involved in the injury response, independent of a metabolic shift. The reciprocal crosstalk between TH/TRs and the immune system has long been discussed, suggesting the modulatory effects of TRs on NF-κB, p38MAPK, and JAK/STAT signaling pathways [86]. Moreover, re-analysis of ChIP-Seq data from mouse hearts revealed that TRα directly regulates several genes associated with inflammation and cell cycle, including *Ccna2*, *Ccnd*, *Il15*, *Il15ra*, *Mdm2*, and *Nfkbia*. These findings suggest a direct regulatory role of TRα in modulating inflammation, cell proliferation, and apoptosis [23, 72].

In addition to TH/TRα signaling axis, hypoxia serves as a key modulator of metabolic adaptation in ischemic myocardial tissue [40]. Particularly, in MI, hypoxia-induced HIF1 regulates myocardial energy metabolism by promoting glycolysis, enabling cells to adapt to the ischemic microenvironment by reducing oxidative stress while maintaining a critical energy supply [87]. Our study focused on HIF3α, which is reported to negatively regulate HIF1-mediated gene expression and contributes to metabolic regulation [44, 88]. In mouse adipocytes, *HIF3α*-driven metabolic reprogramming upregulates thermogenic gene expression, including *Ucp1*, *Elovl3*, *Prdm16*, *Dio2*, and *Ppargc1a* [89]. Notably, *Dio2*, an enzyme that synthesizes active TH (in the form of T3) from thyroxine (T4), links HIF3α to TH signaling. Furthermore, in vitro knockdown of *Hif3α* in neonatal rat CMs reduced cell viability and mitochondrial membrane potential, suggesting a protective role for *Hif3α* in CMs [90]. These findings highlight that HIF3α also regulates metabolism correlated with TH, particularly in terms of thermogenesis and mitochondrial function.

In this study, we found that *hif3a* expression is *thraa*-dependent, with a strong injury-induced upregulation of *hif3a* observed in *thraa*^+/−^ mutants (6-fold increase at 15 dpi). Additionally, ChIP-Seq data from mouse hearts indicate direct binding of TRα to the transcription starting site (TSS) of *Hif3α*. Collectively, we suggest that *hif3a* is a direct downstream target of *thraa* in zebrafish, where it may act as a dominant negative regulator of *hif1aa* and *hif1ab*, and antagonize the hypoxic response upon cardiac injury. Our findings demonstrated that disruption of *hif3a* impairs heart regeneration, as evidenced by a larger fibrotic scar area at 30 dpi and compromised functional recovery, supporting a cardioprotective role for *hif3a* during heart regeneration. However, the metabolic reprogramming in *hif3a* KO mutants during heart regeneration, and the interplay between the TH/TRα signaling axis and HIF3α in this process, remain elusive. Therefore, further investigation is needed to elucidate the relationships among the TH/TRα signaling axis, hypoxia, and metabolic shifts in heart regeneration.

## Conclusions

In conclusion, our study highlights the essential roles of *thraa* and *hif3a* in zebrafish heart regeneration. Moderate reduction in TH/TRα signaling promotes heart regeneration by modulating the inflammatory response and enhancing CM proliferation under distinct metabolic conditions. Additionally, we propose a transcriptional link between *thraa* and *hif3a*, suggesting that the TH/TRα signaling axis interacts with HIF3α to coordinate hypoxic response and metabolic reprogramming during heart regeneration. Their combined roles may serve as a novel therapeutic strategy for treating patients with AMI.

## Methods

### Zebrafish housing and husbandry

WT and transgenic zebrafish lines were housed in animal core facility of The Chinese University of Hong Kong. Zebrafish were maintained at 28.5 °C and pH 7.0-7.5, and under a 14-hour light and 10-hour dark cycle. Zebrafish were fed 3 times a day with dry feeds. All experiments of zebrafish were conducted under the regulation of Animals (Control of Experiments) Ordinance (Cap. 340) of Hong Kong legislation and have been approved by the university’s Animal Experimentation and Ethics Committee (AEEC, ref no.: 21-202-CRF).

### Collection of *thraa* zebrafish mutant line

The *thraa* mutant zebrafish line was a gift from Prof. Sheue-yann Cheng at the National Institute of Health (NIH) in the U.S.A. This transgenic line was generated by CRISPR/Cas9 technology and genotyped as previously described [49].

### Generation of *hif3a* zebrafish mutant line

*hif3a* mutant line was generated by CRISPR/Cas9 technology as we previously described [91–94]. We utilized three pre-designed single-guide RNAs (sgRNAs) available on CRISPRscan website [95]. These sgRNAs target exon 11, 12, and 13 of the *hif3a* gene (ENSDARG00000100826), respectively, covering the transactivation domain (N-TAD) (Supplementary Table S1). Three sgRNAs were synthesized by in vitro transcription using mMESSAGE mMACHINE™ SP6 Transcription Kit (Thermo Fisher Scientific, AM1340), according to manufacturer’s instructions, followed by the purification procedures using RNeasy MinElute Cleanup Kit (QIAGEN, #74204). Then, the purified sgRNAs were co-injected with Guide-it™ Recombinant Cas9 protein (Takara Bio, #632678) to WT zebrafish embryos at one-cell stage according to the previously published protocol [96]. In brief, 1 nL of solution containing Cas9 protein (400pg/nL) and mixed sgRNAs (total 200pg/nL) in a 2:1 ratio was prepared and injected with glass capillary. Injected embryos were raised to adulthood to serve as candidate founders (F0). For founder identification, F0 fishes were screened by T7E1 assay with PCR primers spanning from exon 9 to exon 15 to validate gene knockout as previously described (Supplementary Table 2) [93]. The identified founder was out-crossed with WT to obtain the F1 generation and the F1 zebrafish were raised to adulthood for breeding F2 generation. F1 fish were then screened for mutation by sanger sequencing to confirm sequences of mutated allele. F1 fish carrying the same mutated allele sequences were crossed to obtain F2 generation for heart injury experiments. *hif3a* mutant at F2 were genotyped with specifically designed PRC primers spanning from exon 11 to intron 13 (Supplementary Table 3).

### Cardiac injury experiments and sample collection

Ventricular amputation method was utilized to study heart regeneration in this study. The experimental procedures of ventricular amputation were performed as previously described [16, 97]. In brief, adult zebrafish at 4- to 6-month-old were anesthetized with 0.02% of Tricaine solution (Sigma-Aldrich, E15021) and subsequently transferred to a wet sponge with ventral side up. Zebrafish hearts were exposed by a small incision and ~ 20% of the ventricular apex was resected. Injured zebrafish were then returned to the aquarium system with fresh water and dry feed supplied. Uninjured zebrafish (as the control group) and injured zebrafish at designated time points were euthanized using an overdose of Tricaine solution (400–500 mg/L). Injured zones from injured zebrafish and ventricular apex from uninjured zebrafish were collected by micro-scissor and stored in TRIzol™ Reagent (Thermo Fisher Scientific, #15596026) for total RNA extraction and downstream experiments. Additionally, the entire injured hearts at 7 dpi, 15 dpi, and 30 dpi were collected and fixed for histological analysis of cell proliferation and fibrosis. Similarly, the entire ventricles from adult zebrafish, including WT, *thraa*^+/−^ mutants, and *thraa*^−/−^ mutants, were also collected in this study. Zebrafish ventricles were harvested using micro-scissors and stored in TRIzol™ Reagent. The ventricular tissue was homogenized in TRIzol™ Reagent with a micropestle for total RNA extraction and downstream experiments.

### Tissue RNA extraction and RT-qPCR

Ventricular tissue from at least five zebrafish was pooled together and served as one biological replicate. Total RNA was extracted from the pooled tissue collected in TRIzol™ Reagent as previously described [98]. At least 500 ng of extracted RNA was then reverse-transcribed with GoScript Reverse Transcription kit (Promega, #A5001) or EasyScript^®^ First-Strand cDNA Synthesis SuperMix (TransGen Biotech, AE301-02) according to the manufacturer’s instructions. RT-qPCR reactions were performed with at least 2 independent biological replicates and at least 3 technical replicates using TB Green Premix Ex Taq II (Tli RNase H Plus) (Takara Bio, RR82WR) or SYBR Green (Thermo Fisher Scientific, #25780) on QuantStudio™ 7 Flex Real-Time PCR System (Thermo Fisher Scientific) or the 7900HT Fast Real-Time PCR System (Thermo Fisher Scientific). The relative expression level of tested genes was normalized against *actb1* or *ef1a*. The sequences and information of RT-qPCR primers are listed in Supplementary Table 4.

### Histological staining and image analysis

To examine the histological structure of the zebrafish heart, we performed Masson’s trichrome and hematoxylin and eosin (H&E) staining for heart sections. The collected hearts were fixed in 4% paraformaldehyde (PFA) at 4 °C overnight and then dehydrated with increasing ethanol gradients. Dehydrated hearts were embedded in paraffin for sectioning. The embedded blocks were sectioned into 5 μm using a Rotary Microtome (Leica, RM2235) with a cooling head and MX35 Ultra™ Microtome blade (Epredia, #3053835).

For Masson’s trichrome staining studying fibrosis, de-hydrated slides were de-waxed with xylene and re-hydrated with decreasing ethanol gradients. Re-hydrated slides were immersed in staining solutions in the following order: Weigert’s iron hematoxylin for 10 min, Biebrich scarlet-acid fuchsin solution for 5 min, 1% of phosphomolybdic-phosphotungstic acid for 1 min, aniline blue solution for 5 min. Stained slides were dehydrated with increasing gradients of ethanol again, cleared in xylene, and finally mounted with mounting medium. Images were acquired by Axioscan 7 Microscope Slide Scanner (ZEISS). Measurement of the fibrotic area was conducted with ImageJ software (v.1.54d). To minimize variability in the fibrotic scar among individual hearts, we examined the slides batch by batch and selected those with the largest fibrotic area. For each biological replicate, at least eight sections were chosen to calculate the average fibrotic area. The averaged fibrotic area was then normalized to the entire ventricular area. Finally, at least five independent biological replicates were used in both *thraa* and *hif3a* experiments. To minimize potential bias, we avoided re-selecting sections or slides from the same sample for analysis, and personnel conducting image analysis were blinded to the genotypes of the samples.

For H&E staining studying cardiac structure, sectioned slides were de-waxed and re-hydrated, as mentioned above. Re-hydrated slides were immersed and stained in hematoxylin for 10 min and eosin for 1 min. Stained slides were dehydrated, cleared, and mounted following the procedures mentioned above. Images were acquired by ECLIPSE Ni-E upright light microscope (Nikon).

### Immunofluorescence staining and image analysis

De-hydrated slides were prepared, de-waxed, and rehydrated as described above. Re-hydrated slides were then immersed in citrate solution and microwaved for ~ 20 min for antigen retrieval. After that, the slides were fixed with 4% of PFA for ~ 15 min, washed with 1x PBS three times for ~ 5 min each, and permeabilized with 0.1% of Triton™ X-100 for ~ 10 min (Sigma Aldrich, T8787). Permeabilized slides were subsequently blocked in 4% of normal goat serum for 1 h. Serum-blocked slides were incubated with primary antibodies prepared in blocking solution, including mouse anti-α-actinin (Santa Cruz Biotechnology, sc-166524, 1:500 dilution), rabbit anti-phospho-histone H3 (Cell Signaling Technology, #3377, 1:500 dilution), at 4 °C overnight. Incubated slides were washed with 1x PBS-Tween 20 three times for ~ 5 min each. Slides were incubated with corresponding secondary antibodies, including goat anti-mouse IgG Alexa Fluor 488 (Thermo Fisher Scientific, A32723, 1:1000 dilution) and goat anti-rabbit IgG Alexa Fluor 555 (Thermo Fisher Scientific, A32732, 1:1000 dilution). After incubating with secondary antibodies, slides were washed with 1x PBS-Tween 20 three times for ~ 5 min each and then incubated with DAPI (Sigma Aldrich, #28718-90-3, 1 µg/ml) at room temperature for ~ 15 min before mounting. Images of IF-stained slides were acquired by TCS SP8 confocal microscope (Leica). Measurement of CM proliferative rate was conducted with ImageJ software (v.1.54d). In brief, a 200 μm × 200 μm square (area: 40,000 μm²) was first defined as the quantification area at the injury site on each section to standardize the analysis area. Then, the α-actinin signal indicating myocardium was used to identify CMs and non-CMs; only α-actinin^+^ cells were counted as CMs. The DAPI marker was used to confirm the true cellular identity of the signal. pHH3^+^ DAPI^+^ CMs were counted and normalized to DAPI^+^ only CMs in the same α-actinin^+^ field. Only sections with over 200 cm counted were considered, and at least 6 sections were counted in each individual heart. Three independent biological replicates were used in this experiment. To minimize potential bias, we avoided re-selecting sections or slides from the same sample for analysis, and personnel conducting image analysis were blinded to the genotypes of the samples.

### EdU assay, staining, and image analysis

Before EdU staining, injured zebrafish were intraperitoneally injected with EdU (Thermo Fisher Scientific, A10044, 15 µL of 100 mM per animal) daily for three days starting from 5 dpi. Zebrafish hearts were collected at 7 dpi and fixed in 4% PFA at 4 °C overnight. Heart section slides were prepared as described above, including dehydration, embedding, sectioning, and antigen retrieval. Sectioned slides were then incubated with Alexa Fluor 647 Azide (Thermo Fisher Scientific, A10277) according to the manufacturer’s instructions. Slides were counterstained with primary antibody for α-actinin, secondary antibody for fluorescence signal and DAPI as described above. The procedures for acquiring IF-stained images and quantification of proliferating CMs are the same as above; EdU^+^ DAPI^+^ CMs were measured as proliferating CMs. Three independent biological replicates were used in this experiment.

### Bulk RNA-Seq and bioinformatic analysis

Two biological replicates of extracted RNA were prepared for each time point and genotype. Extracted RNA was sent to Novogene Ltd. (Beijing, China) for library construction and bulk RNA sequencing. Constructed libraries were sequenced on NovaSeq 6000 PE150 system, and ~ 6 GB of raw data was collected from each sample.

QC and adapters trimming were performed by Trim Galore (v.0.6.10) and aligned with the reference genome of zebrafish (Genome assembly of *Danio Rerio*: GRCz11) using STAR (v2.7.9) [99, 100]. The output files in BAM format were passed to RSEM (v1.3.3) for quantifying transcripts [101]. DESeq2 (v1.42.0) was used for differential expression analysis [102]. In the heart development study, genes with|log2FC| ≥ 0.585 and an adjusted *p*-value < 0.05 were considered differentially expressed genes (DEGs). In contrast, for the heart regeneration study, genes with|log2FC| ≥ 1 and an adjusted *p*-value < 0.05 were considered as DEGs. Enrichment of Gene Ontology (GO), pathway analysis, motif analysis, ligand-receptor interaction, protein-protein interaction network (PPI), and Gene Set Enrichments Analysis (GSEA) were conducted by clusterProfiler (v.4.10.0), HOMER (v4.11), DanioTalk (v.2.0.1), STRING database, and GSEA software (v.4.1.0) [103–107]. Mitochondrial and metabolic genes used in this study were referred from the mitoXplorer 2.0 database [57].

### In vivo electrocardiogram (ECG) of adult zebrafish

Electrocardiogram (ECG) was performed on uninjured and injured adult zebrafish using the iWorx IX-100 F Zebrafish ECG Recording and Analysis System. Adult zebrafish were anesthetized in 0.02% of Tricaine for 4 to 6 min until the equilibrium and muscle tone were lost. Anesthetized zebrafish were then placed on the provided plastic holder with the ventral side up. ECG lead electrodes with electrode gel were placed onto zebrafish skin at the location of heart. The positioning of the electrodes was adjusted until an ECG signal was observed. For data acquisition, the ECG signal was recorded for at least 1 min. The collected data was then analyzed using the LabScribe software (v.24.0) (iWorx Inc). Filtered signals from every five cycles were analyzed using the default settings of the software. Mean values of cycles of individual zebrafish were presented. Data sets from WT were repeatedly used in the *thraa* and *hif3a* experiments.

### Statistical analysis

Statistical analyses were performed in GraphPad Prism (v.5.0). Statistical significance was calculated by unpaired Student’s *t*-test, unpaired *t*-test with Welch’s test, log-rank test, or one-way ANOVA followed by post-hoc Bonferroni correction as indicated in the figures. Error bars in figures indicate mean ± s.e.m.

## Electronic supplementary material

Below is the link to the electronic supplementary material.

Supplementary Material 1

Supplementary Material 2

Supplementary Material 3

Supplementary Material 4

Supplementary Material 5

Supplementary Material 6

Supplementary Material 7

Supplementary Material 8

Supplementary Material 9

Supplementary Material 10

Supplementary Material 11

Supplementary Material 12

Supplementary Material 13

Supplementary Material 14

Supplementary Material 15

Supplementary Material 16

Supplementary Material 17

Supplementary Material 18

Supplementary Material 19

Supplementary Material 20

Supplementary Material 21

## Data Availability

The datasets supporting the conclusions of this article are available in the Gene Expression Omnibus (GEO) repository. Raw sequencing and processed data were deposited at https://www.ncbi.nlm.nih.gov/geo/query/acc.cgi?acc=GSE282482.
