# Supplementary material for: Knockout of thyroid hormone receptor alpha a (thraa) enhances cardiac regeneration in zebrafish through metabolic and hypoxic regulation
Source: Cell Commun Signal. 2025 Jul 16;23:340. doi: 10.1186/s12964-025-02350-5 (PMC12265366; doi:10.1186/s12964-025-02350-5)
Supplement: Supplementary file 15 — Supplementary Material 15 [file 12964_2025_2350_MOESM15_ESM.docx]

Table S1. Sequences of sgRNAs for generating *hif3a* zebrafish mutant line

| **Targeted exon** | **Locus** | **Strand** | **Target sequences** |
| --- | --- | --- | --- |
| 11 | 15:25508677-25508700 | - | GAGGAGAGCCTAACCCCGAAAGG |
| 12 | 15:25508480-25508503 | - | GAGTGAAATAACACCACCTTTGG |
| 13 | 15:25507878-25507901 | + | GAGCAGAAGCTGGTCCTCTATGG |
