# Supplementary material for: Knockout of thyroid hormone receptor alpha a (thraa) enhances cardiac regeneration in zebrafish through metabolic and hypoxic regulation
Source: Cell Commun Signal. 2025 Jul 16;23:340. doi: 10.1186/s12964-025-02350-5 (PMC12265366; doi:10.1186/s12964-025-02350-5)
Supplement: Supplementary file 16 — Supplementary Material 16 [file 12964_2025_2350_MOESM16_ESM.docx]

Table S2. Sequences of PCR primers for screening *hif3a* zebrafish founders (F0)

| **Direction** | **Primer sequences (5’ to 3’)** |
| --- | --- |
| F | GCAGCACTGCCAAACTCTTC |
| R | TCCTCCTGTGTTTTGTGCGT |
