# Supplementary material for: Knockout of thyroid hormone receptor alpha a (thraa) enhances cardiac regeneration in zebrafish through metabolic and hypoxic regulation
Source: Cell Commun Signal. 2025 Jul 16;23:340. doi: 10.1186/s12964-025-02350-5 (PMC12265366; doi:10.1186/s12964-025-02350-5)
Supplement: Supplementary file 17 — Supplementary Material 17 [file 12964_2025_2350_MOESM17_ESM.docx]

Table S3. Sequences of PCR primers for genotyping *hif3a* zebrafish mutant

| **Direction** | **Primer sequences (5’ to 3’)** | **Ratio** |
| --- | --- | --- |
| F | ATGGAGATGGAAGGAGTGGAGA | 0.5 |
| F | TTGGCTGCTAGATGGGACAAG | 0.5 |
| R | TTGCACCTTCACATCGTTTTGATT | 1 |
