# Supplementary material for: Knockout of thyroid hormone receptor alpha a (thraa) enhances cardiac regeneration in zebrafish through metabolic and hypoxic regulation
Source: Cell Commun Signal. 2025 Jul 16;23:340. doi: 10.1186/s12964-025-02350-5 (PMC12265366; doi:10.1186/s12964-025-02350-5)
Supplement: Supplementary file 18 — Supplementary Material 18 [file 12964_2025_2350_MOESM18_ESM.docx]

Table S4.List of RT-qPCR primers

| **Types of primers** | **Targeted gene** | **Direction** | **Primer sequences(5’ to 3’)** | **Remarks** |
| --- | --- | --- | --- | --- |
| Reference | *actb1* | F | CGAGCAGGAGATGGGAACC |  |
|  |  | R | CAAGATTCCATACCCAGGAAGGA |  |
|  | *ef1a* | F | GCCGTCCCACCGACAAG |  |
|  |  | R | CCACACGACCCACAGGTACAG |  |
| Inflammation | *il1b* | F | CTGAAATGATGGCATGCGGG |  |
|  |  | R | TGCAAGCGGATCTGAACAGT |  |
|  | *tnfa* | F | AGGAGAGTTGCCTTTACCGC |  |
|  |  | R | TATGGAGCGTGAAGCAGACG |  |
|  | *il6* | F | ATGACGGCATTTGAAGGGGT |  |
|  |  | R | TCAGGACGCTGTAGATTCGC |  |
|  | *nfkbiaa* | F | ATGAGACGCGGGAGATTTCG |  |
|  |  | R | TCCGTCCTCGGTGACTACTT |  |
|  | *nfkbiab* | F | TATAATGACGACGGTCGCGG |  |
|  |  | R | GCGACGGAAGATTGTCCTCT |  |
|  | *nfkb1* | F | GAACACCATGGACCCCACAA |  |
|  |  | R | GCAAGGCCCATCAACTGTTC |  |
|  | *nfkb2* | F | CCAGAAAACTGTTGTGGCGG |  |
|  |  | R | GGAGCCTGACTGAGCTTGTT |  |
| Cell cycle | *ccna2* | F | GAATCGGCTTTTGTACGCGG |  |
|  |  | R | CAGCTCAAAACGCCCAGAGA |  |
|  | *ccnb1* | F | GGAACATGCGTGCCATTCTT |  |
|  |  | R | ACATGGCCGTTACACCAACA |  |
|  | *ccnb2* | F | AACTTCAGCTGGTGGGTGTC |  |
|  |  | R | TCCGTAAGAAGTGCAGAGGC |  |
|  | *cdc3a* | F | TCGAGCATTAAGCAGCAAACAG |  |
|  |  | R | AAACACAGGCCCAACTGACT |  |
|  | *e2f1* | F | TACAGCTACGGCTCCTCACT |  |
|  |  | R | CCTTTGGAGCTCTTCAGCGA |  |
| CM differentiation | *myh6* | F | ACGCGCAACAACTTGAAGAC |  |
|  |  | R | AACACTTTGCATTCACCGCC |  |
|  | *myh7* | F | GACAAGGCAATCATGGGGGA |  |
|  |  | R | TGTCACCTTCACGACTGACG |  |
|  | *tnni1b* | F | TTCTCTGACCATCCGAGCAC |  |
|  |  | R | AGCTCAGCAAAGGACATCCC |  |
|  | *tnni1c* | F | ATGGCAGACGACGATACACC |  |
|  |  | R | TTGAGGTTTGCTCGGAGGTC |  |
|  | *tnnt2a* | F | GAGTCAGTGACCATCAGAAAACG |  |
|  |  | R | CAACAGTGGTCAGCTCCTCTC |  |
| Hypoxia | *hif1aa* | F | AGCCGCCACACTTTAGACAT |  |
|  |  | R | CCTCTGGATCAAAACCCAAG |  |
|  | *hif1ab* | F | GCCACACTCTGGACATGAAG |  |
|  |  | R | TCAAGAGGTCATCTGGCTCA |  |
|  | *hif2aa* | F | CATGAGCAGACATAGTATGGATATGA |  |
|  |  | R | CAAGAAGTTCCTCGGGTCTG |  |
|  | *hif2ab* | F | TCTTCAGGATTGTAGCCCATC |  |
|  |  | R | CCACAGCATGGACATGAAGTAT |  |
|  | *hif3a* | F | CTGCCTCAACTTCATTCTC | 1^st^ pair |
|  |  | R | AGTCTTCTTGCTCCTCCTTA |  |
|  |  | F | ACCAGTAATTCTAGGAAACGGTGT | 2^nd^ pair |
|  |  | R | CATCCAGTAATGTGTGGCTGAG |  |

- The pair of *ef1a* primers, used as a reference, was only employed in the experiment of Supplementary Figure S8B.
- Two pairs of *hif3a* primers were used in this manuscript. The first pair of *hif3a* primers was used in the experiment for Figure 6B. The second pair of *hif3a* primers was used in the experiment of Supplementary Figure S8B.
