# Supplementary figures and images for: Knockout of thyroid hormone receptor alpha a (thraa) enhances cardiac regeneration in zebrafish through metabolic and hypoxic regulation
Source: Cell Commun Signal. 2025 Jul 16;23:340. doi: 10.1186/s12964-025-02350-5 (PMC12265366; doi:10.1186/s12964-025-02350-5)

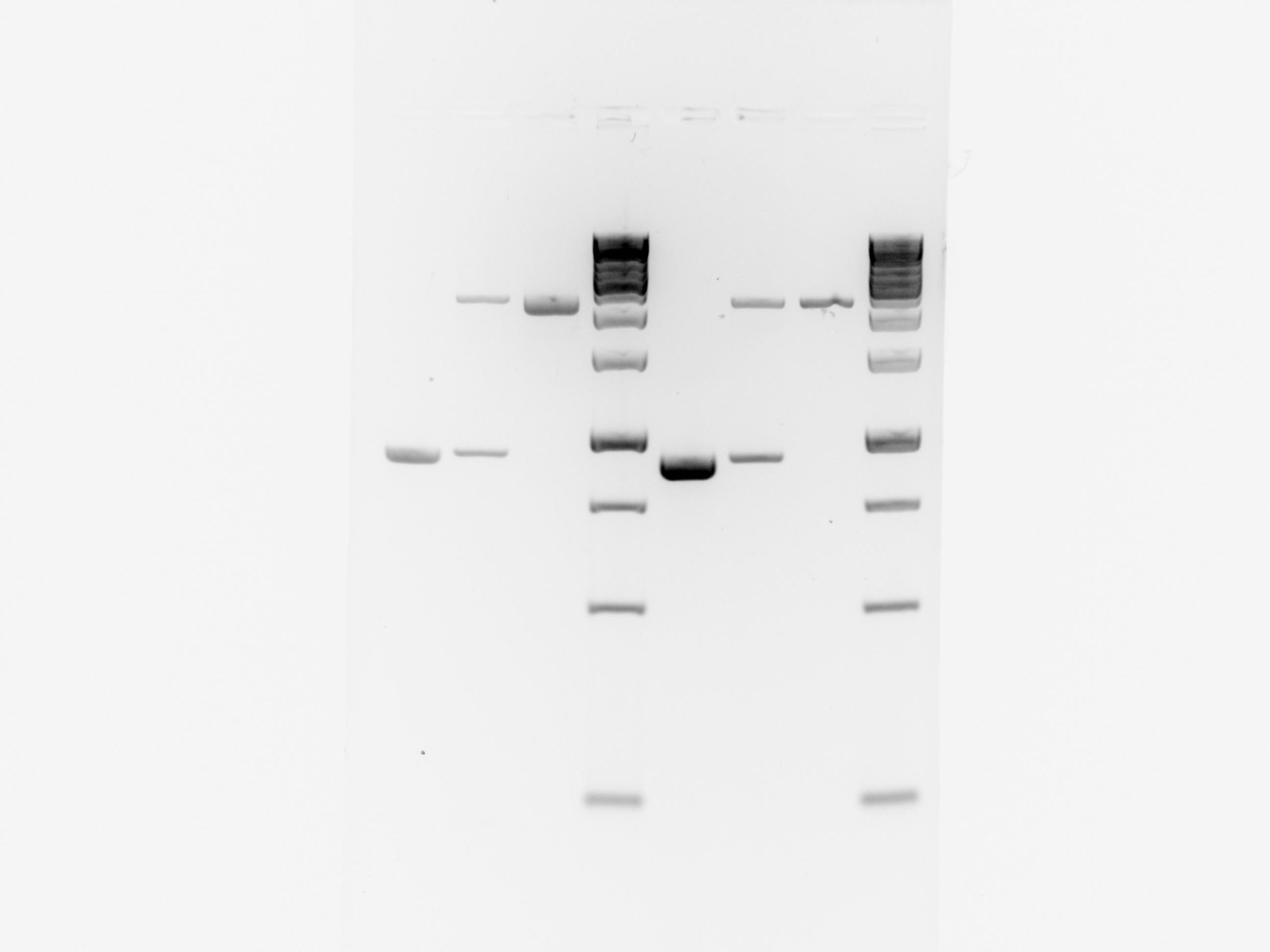

Supplement: Supplementary file 19 — Supplementary Material 19 [file 12964_2025_2350_MOESM19_ESM.jpg]

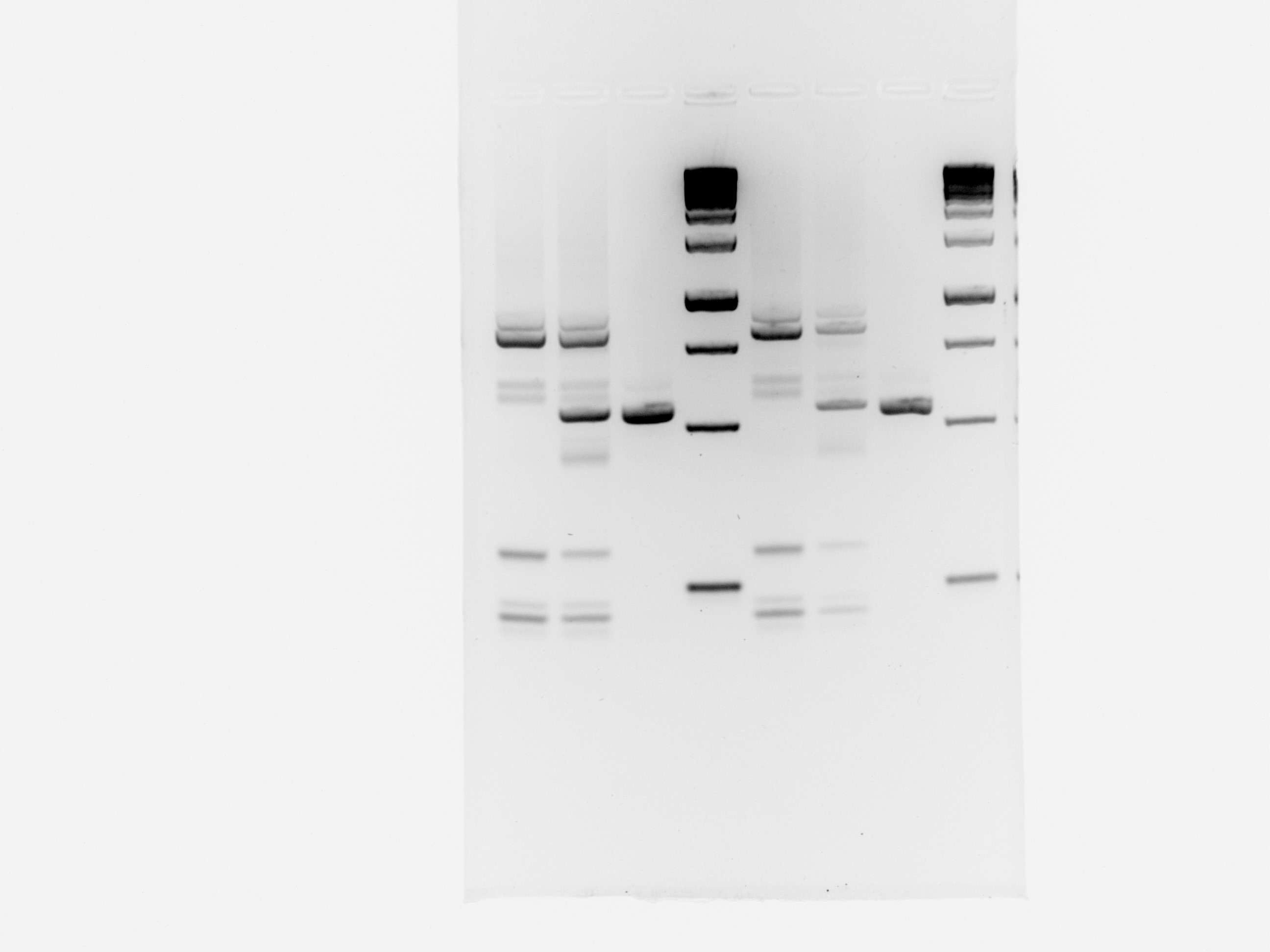

Supplement: Supplementary file 20 — Supplementary Material 20 [file 12964_2025_2350_MOESM20_ESM.jpg]
