# Supplementary material for: Knockout of thyroid hormone receptor alpha a (thraa) enhances cardiac regeneration in zebrafish through metabolic and hypoxic regulation
Source: Cell Commun Signal. 2025 Jul 16;23:340. doi: 10.1186/s12964-025-02350-5 (PMC12265366; doi:10.1186/s12964-025-02350-5)
Supplement: Supplementary file 21 — Supplementary Material 21 [file 12964_2025_2350_MOESM21_ESM.docx]

Supplementary Figure S1

**C**

**B**

**A**


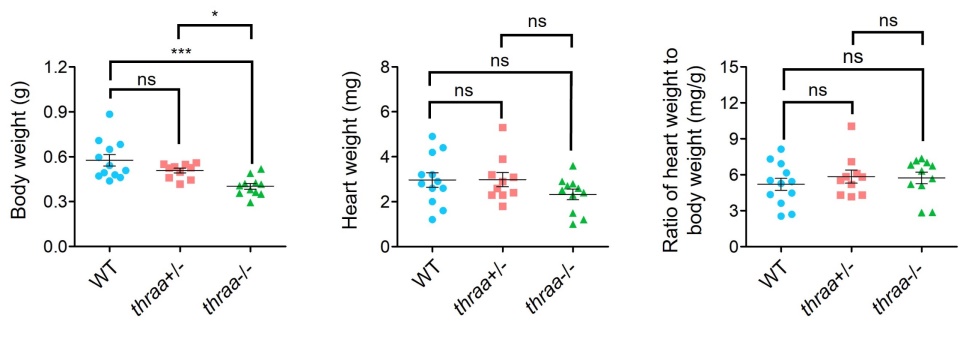

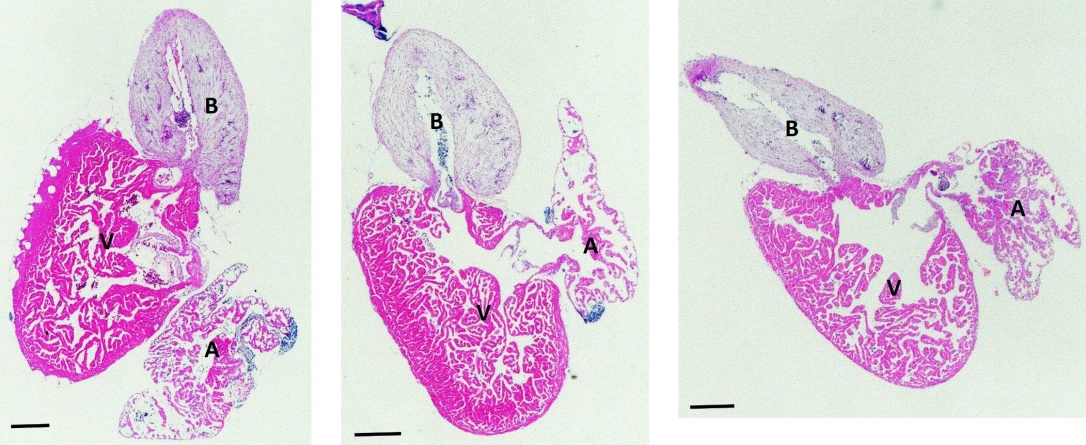

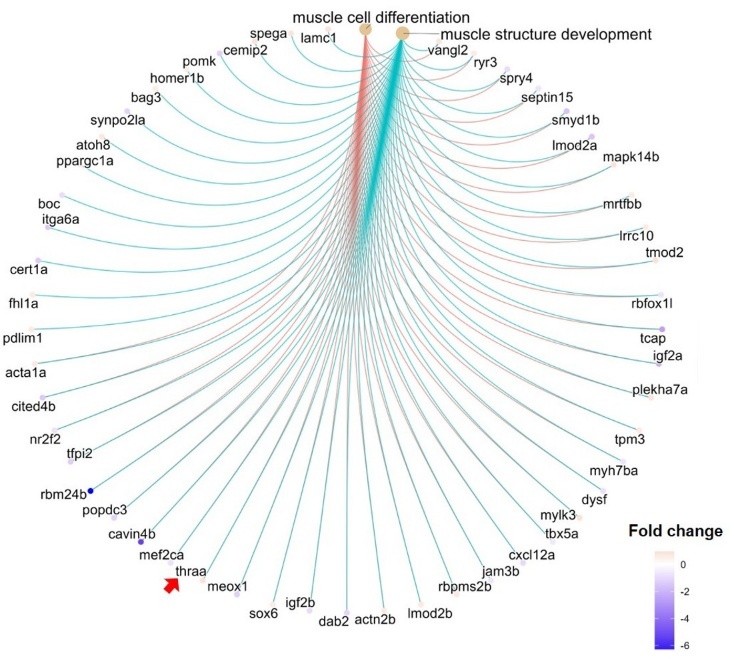

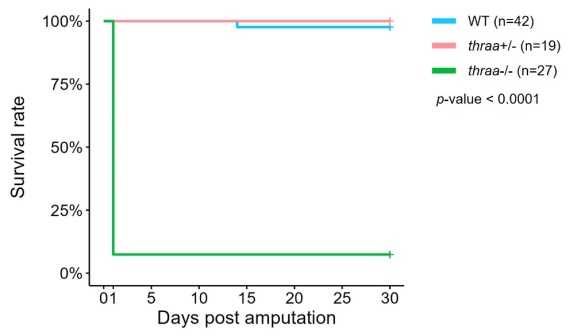

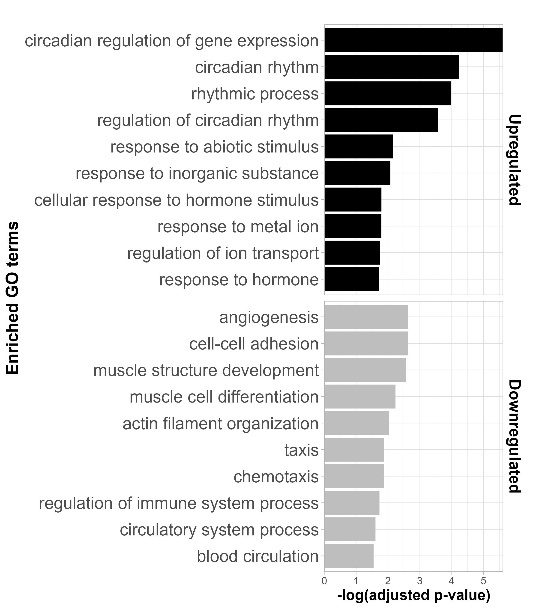


**D**

**E**

**F**

**H**

**G**

**I**

Supplementary Figure S1. Knockout of *thraa* leads to developmental defect and differential response to heart injury.

**A-C)** Measurements of the physiological parameters in adult WT and *thraa* mutants. Body weight **A)**, heart weight **B)**, and ratio of body weight to heart weight **C)**. *n* = 12 (WT), 10 (*thraa*^+/−^ mutants) and 11 (*thraa*^−/−^ mutants). Results are shown in mean ± s.e.m;
ns, *P* > 0.05; * *P* < 0.05; *** *P* < 0.001 by one-way ANOVA followed by Bonferroni’s test.

**D-F)** H&E staining of uninjured heart sections from adult WT and *thraa* mutants, including WT **D)**, *thraa*^+/−^ mutants **E)**, and *thraa*^−/−^ mutants **F)**. A, atrium; V, ventricle; BA, bulbus arteriosus. Scale bar, 500 µm.

**G)** Enriched GO terms in upregulated (upper panel) and downregulated (lower panel) DEGs by *thraa*^−/−^ mutants vs. WT.

**H)** Circular net plot shows DEGs in selected enriched GO terms related to muscle development. Dot color indicates the fold change of DEGs by *thraa*^−/−^ mutants vs. WT.

**I)** Survival curves of WT and *thraa* mutants after cardiac surgery. *n* = 42 (WT), 19 (*thraa*^+/−^ mutants) and 27 (*thraa*^−/−^ mutants). Statistical significance of survival rate was calculated by log-rank test.

Supplementary Figure S2


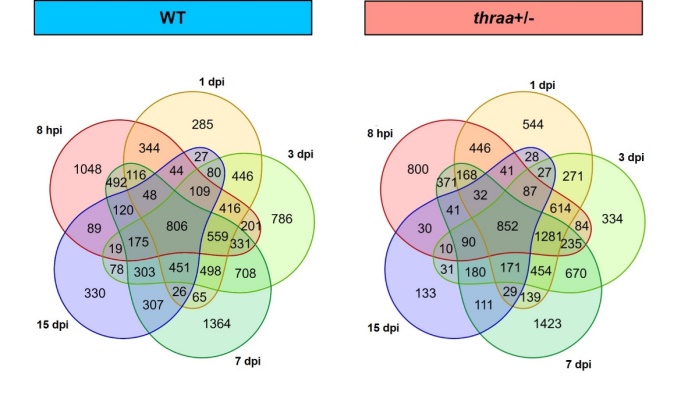

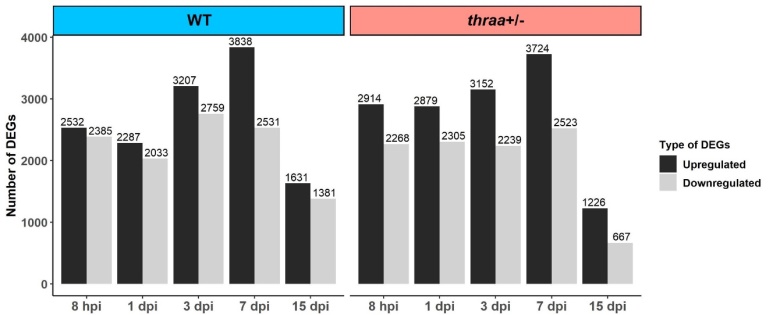

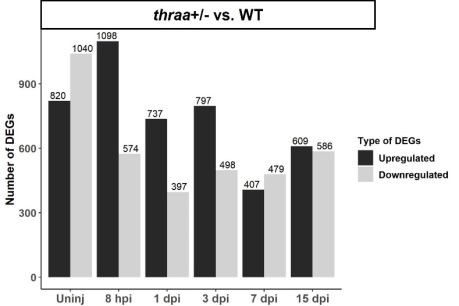


**B**

**C**

**A**


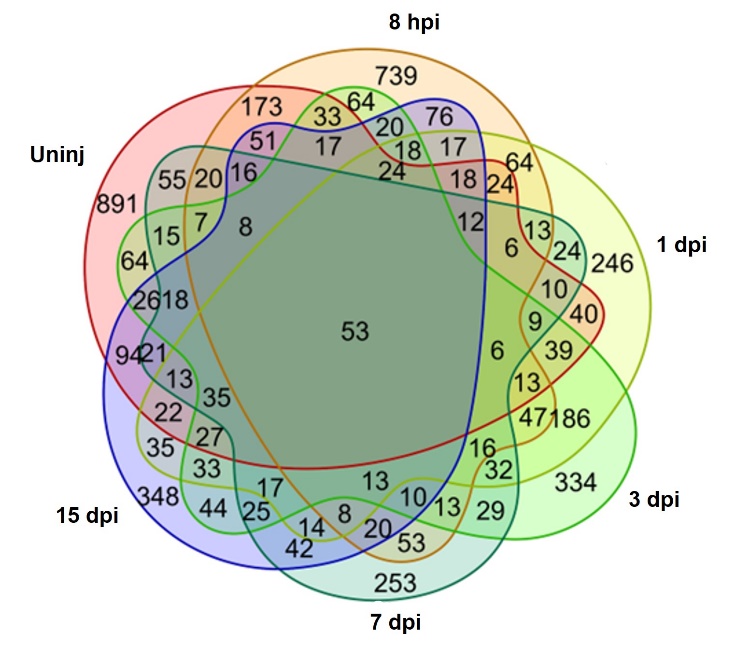

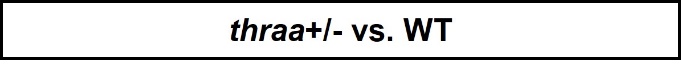


**D**

Supplementary Figure S2. Statistics of the DEGs identified in both WT and *thraa*^+/−^ mutants.

1. Bar plot shows the statistics of identified DEGs obtained from both WT and *thraa^+/−^* mutants through time-course analysis.
2. Venn diagrams of DEGs obtained from both WT and *thraa^+/−^* mutants through time-course analysis.
3. Bar plot shows the statistics of identified DEGs obtained by *thraa*^+/−^ mutants vs. WT (per-time point analysis).
4. Venn diagram of DEGs obtained by *thraa*^+/−^ mutants vs. WT (per-time point analysis).

Supplementary Figure S3


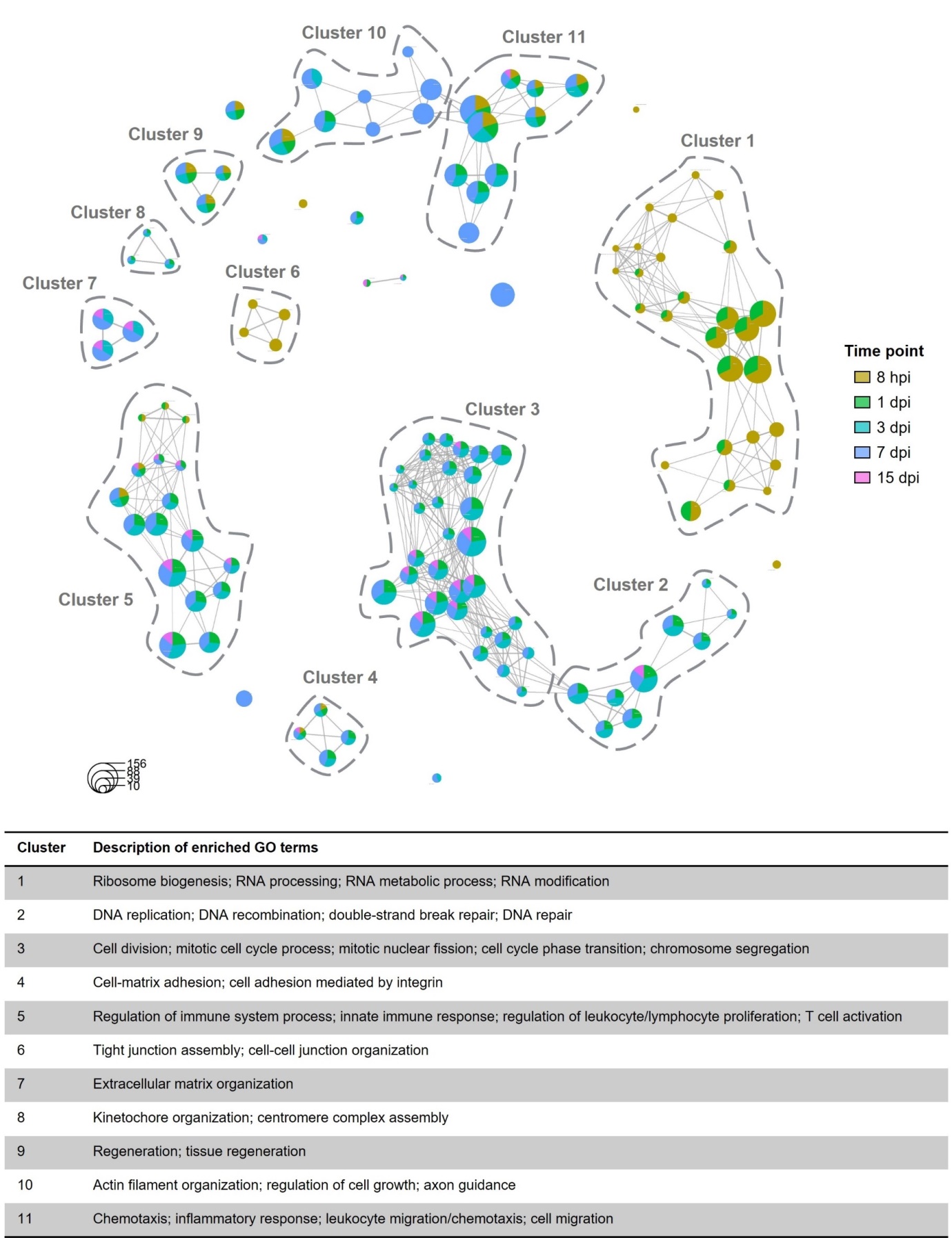


**Upregulated GO terms in biological process in WT**

**A**

Supplementary Figure S3

**B**


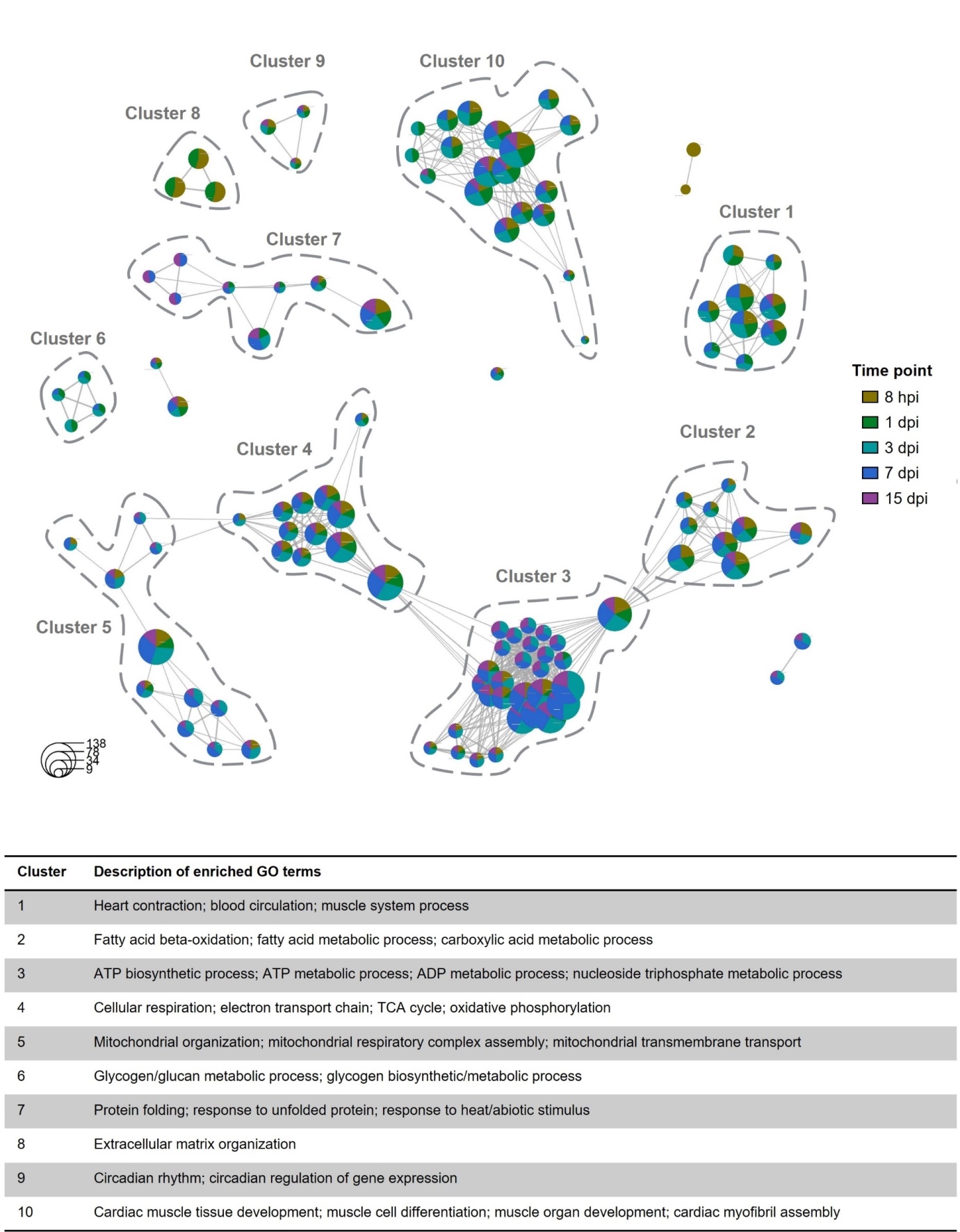


Supplementary Figure S3
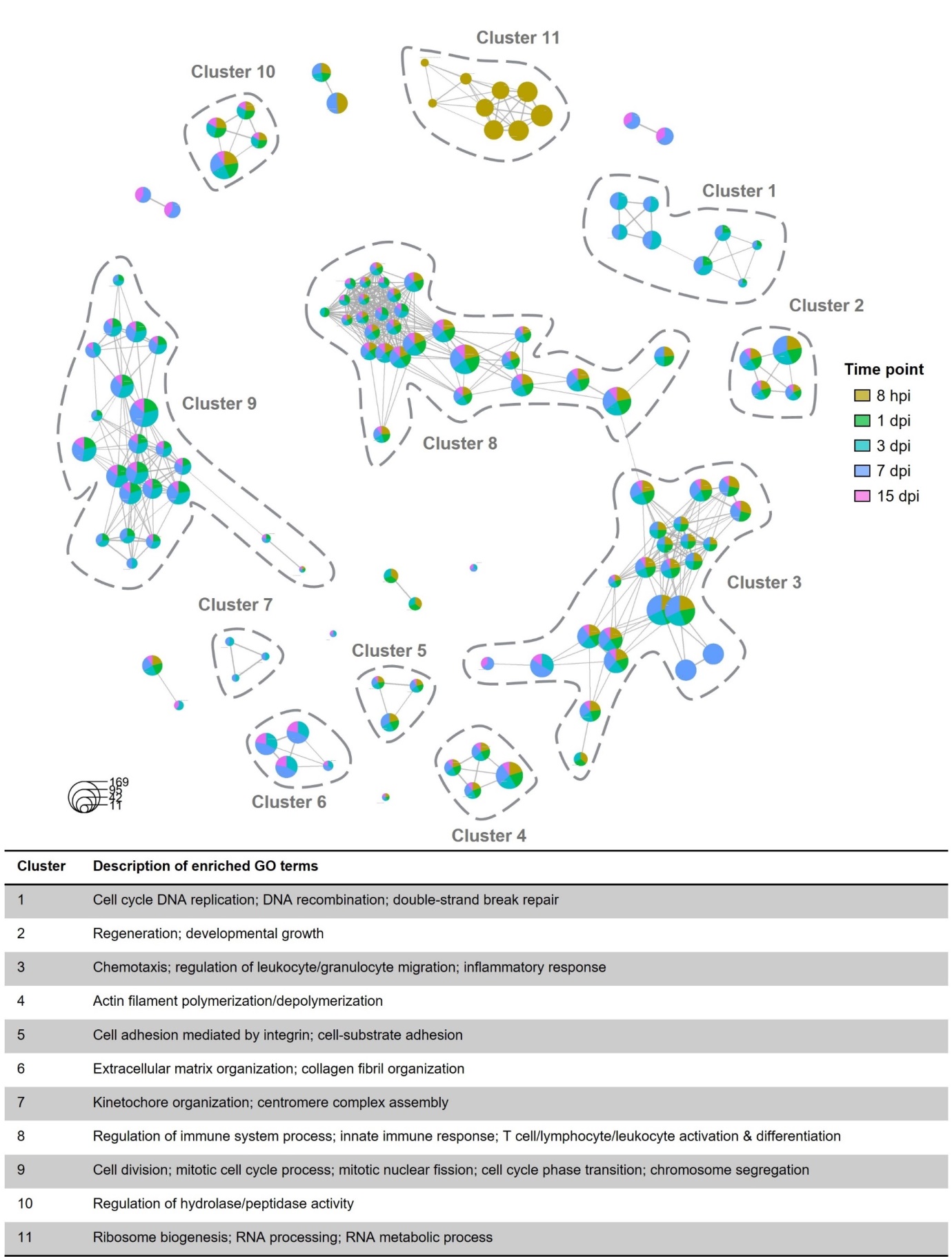


**C**

Supplementary Figure S3


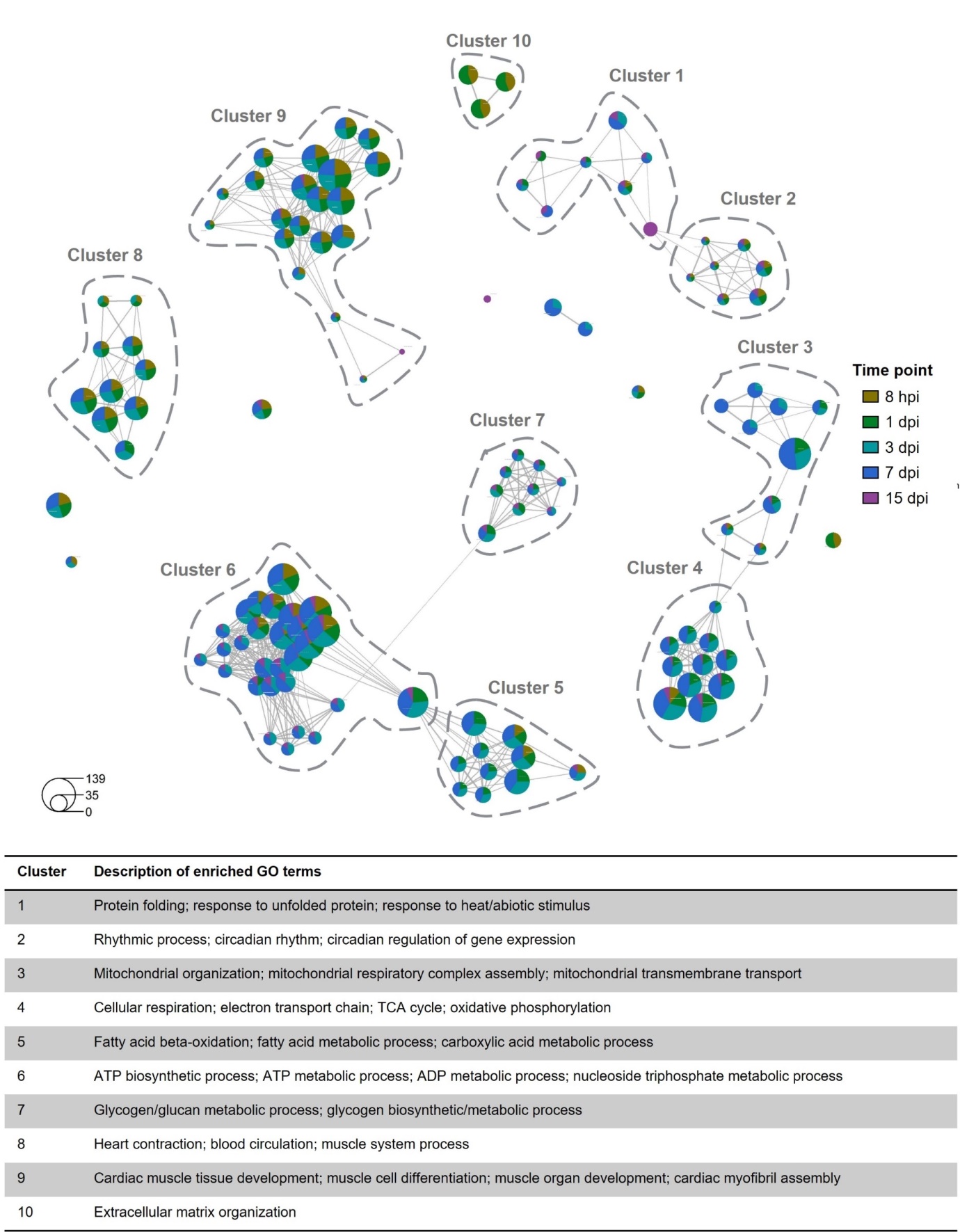


**D**

Supplementary Figure S3. GO term enrichment analysis of both WT and *thraa*^+/−^ mutants.

**A-D)** Clustering of enriched GO terms in biological processes of both WT and *thraa^+/−^* mutants through time-course analysis. Enriched GO terms for upregulated **A)** and downregulated **B)** DEGs in WT, and upregulated **C)** and downregulated **D)** DEGs in *thraa*^+/−^ mutants. Enriched GO terms (adjusted *p*-value < 0.05) are clustered according to their similarities and described in the table below. The circle size indicates the number of genes in each GO term; slices in the pie chart represent the ratio of genes.

Supplementary Figure S4

**A**


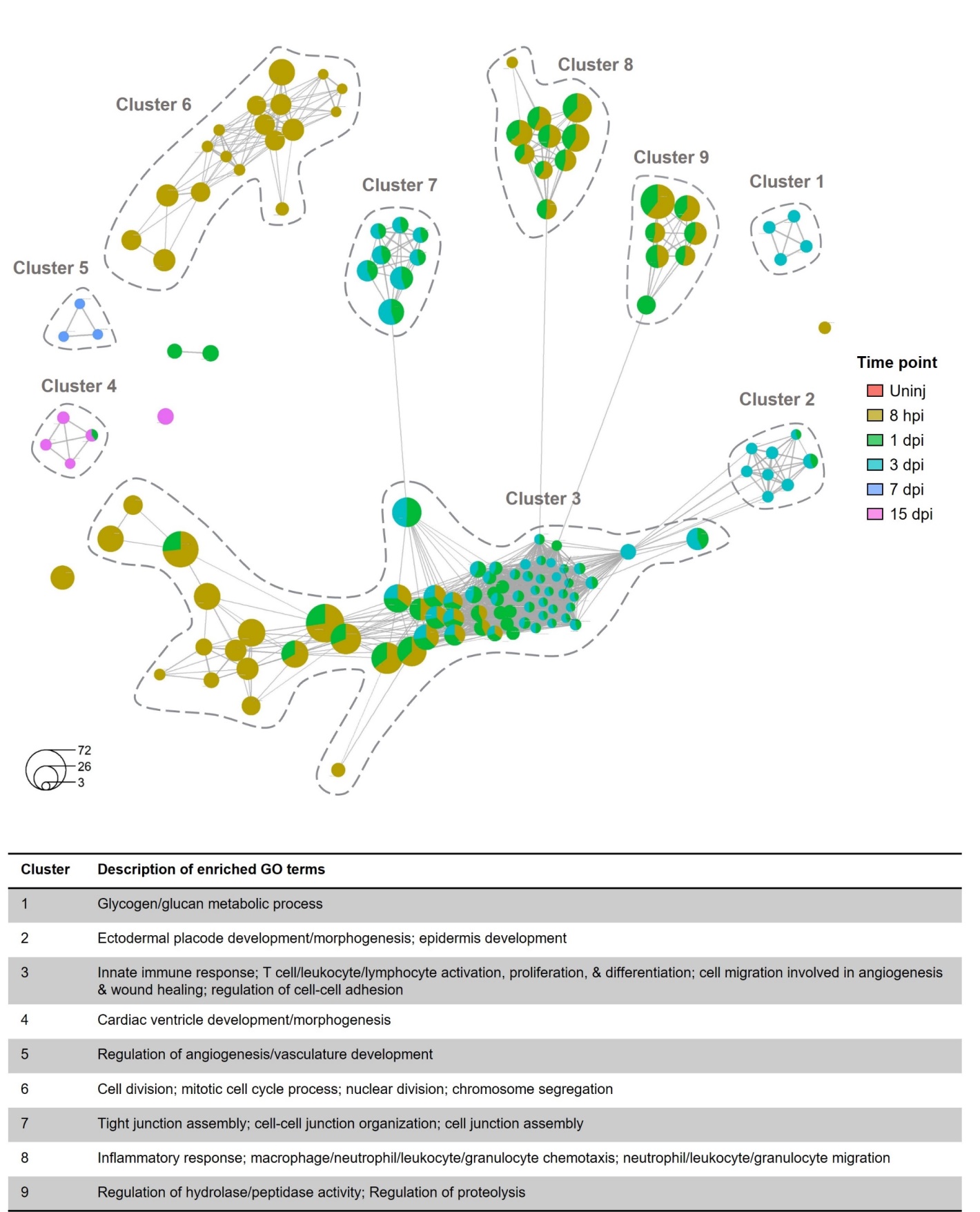


Supplementary Figure S4


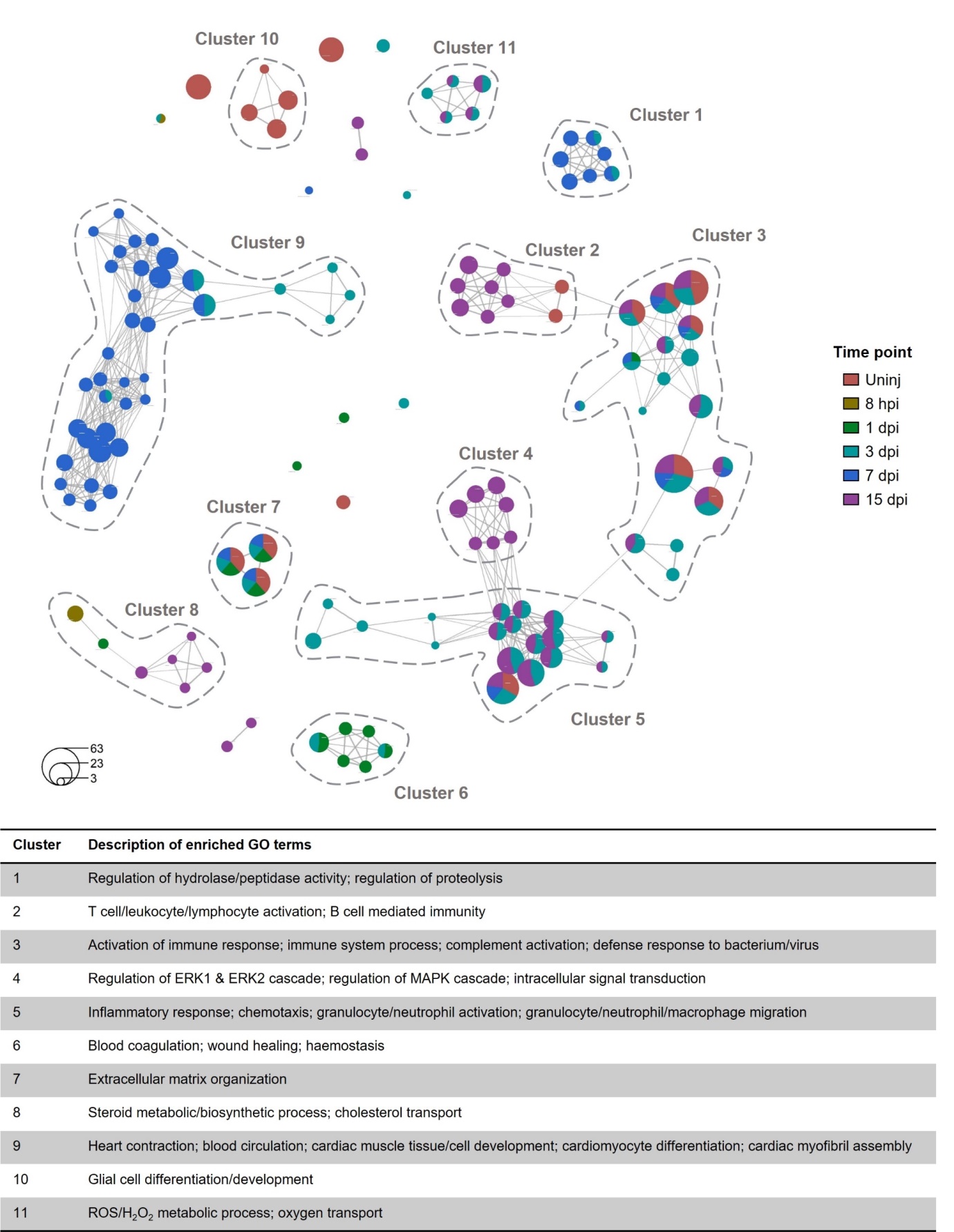


**B**

Supplementary Figure S4. GO term enrichment analysis by *thraa*^+/−^ mutants vs. WT.

**A-B)** Clustering of enriched GO terms in biological processes for upregulated **A)** and downregulated **B)** DEGs by *thraa^+/−^* mutants vs. WT. Enriched GO terms (adjusted
*p*-value < 0.05) are clustered according to their similarities and described in the table below. The circle size indicates the number of genes in each GO term; slices in the pie chart represent the ratio of genes.

Supplementary Figure S5


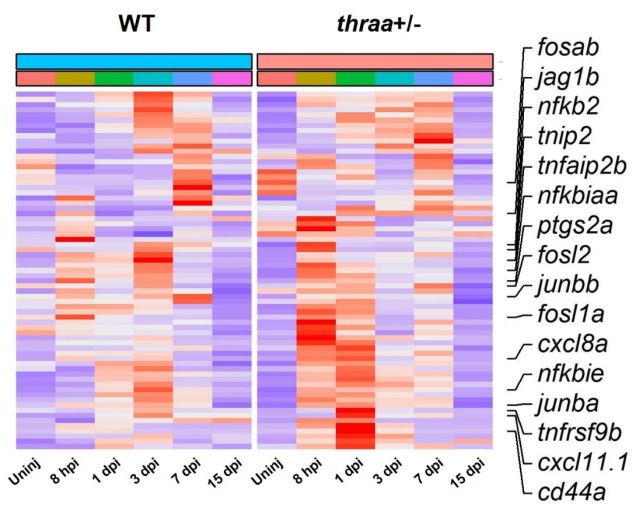

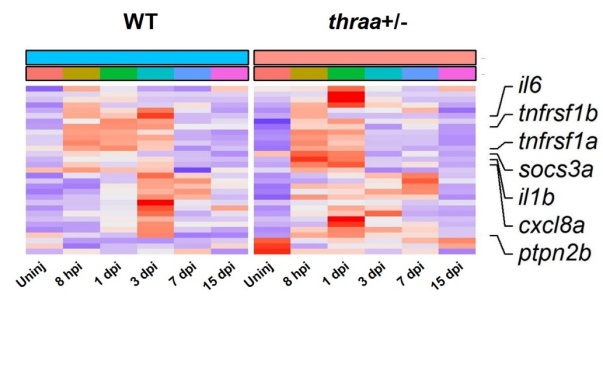

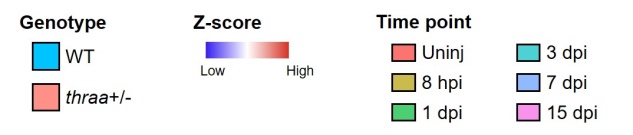


**A**

**B**

Supplementary Figure S5. Heatmaps of significantly upregulated HALLMARK gene sets in inflammatory response.

**A-B)** Heatmaps show the expression of HALLMARK gene sets in TNFα signaling via NF- κB **A**) and IL-6/JAK/STAT3 signaling **B**).

Supplementary Figure S6
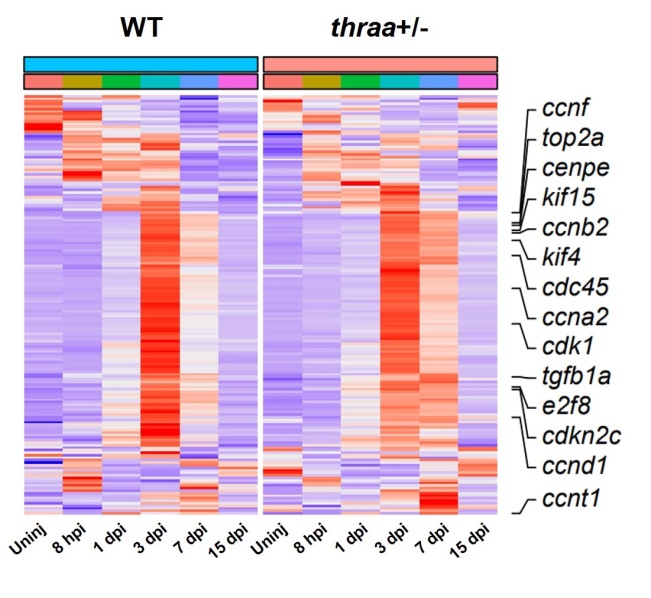

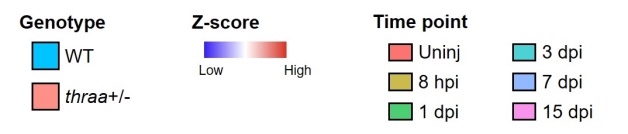

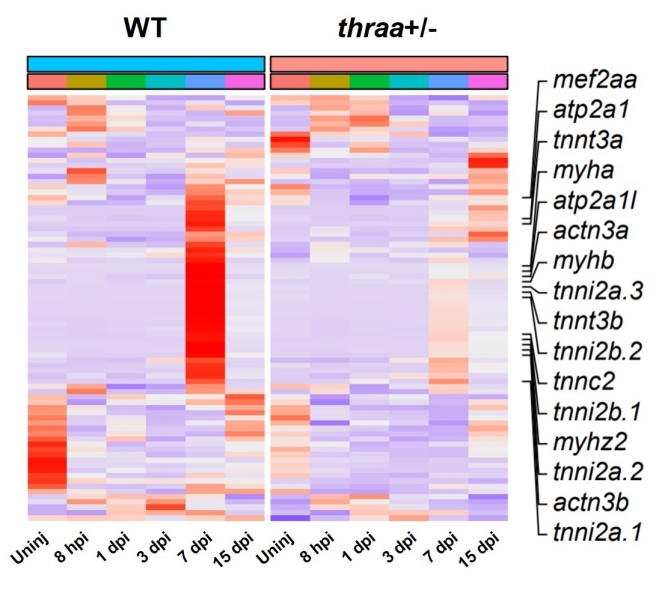


**B**

**A**

Supplementary Figure S6. Heatmaps of significantly altered HALLMARK gene sets at 7 dpi.

**A-B)** Heatmaps show the expression of genes in GSEA as shown in Fig. 3B and 4B. Genes belonged to G2M and E2F gene sets **A)**, and myogenesis **B)** are shown.

Supplementary Figure S7


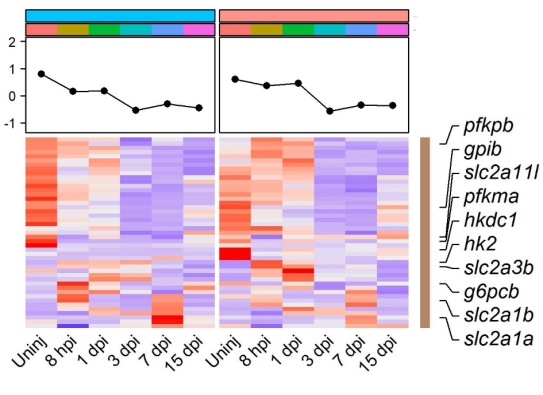

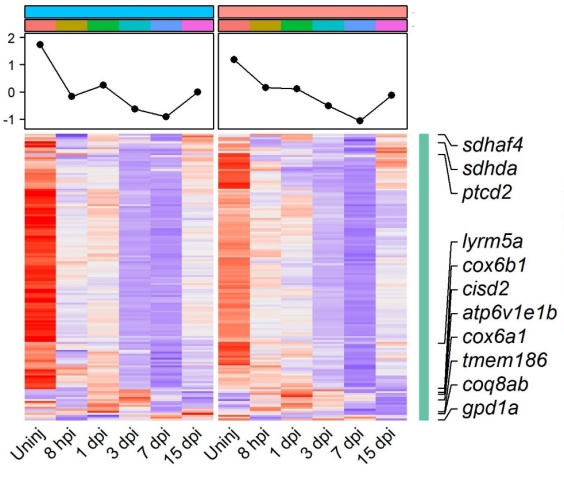

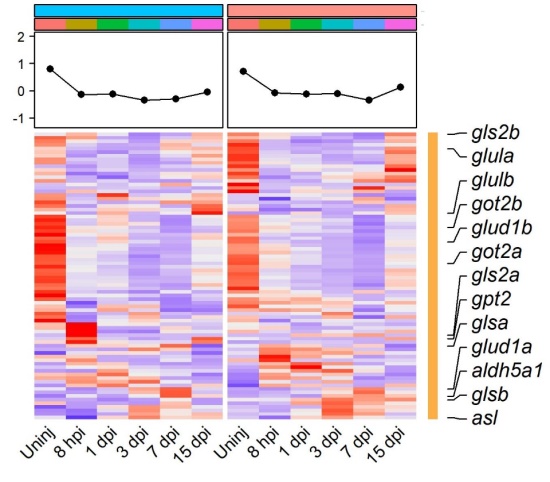

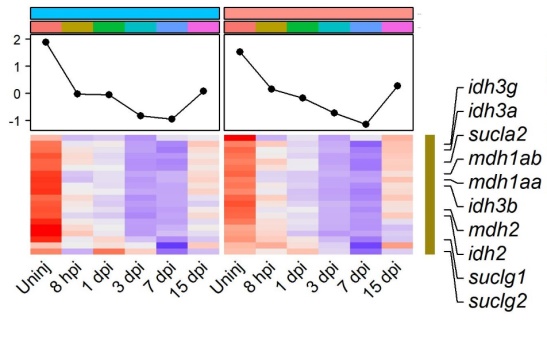

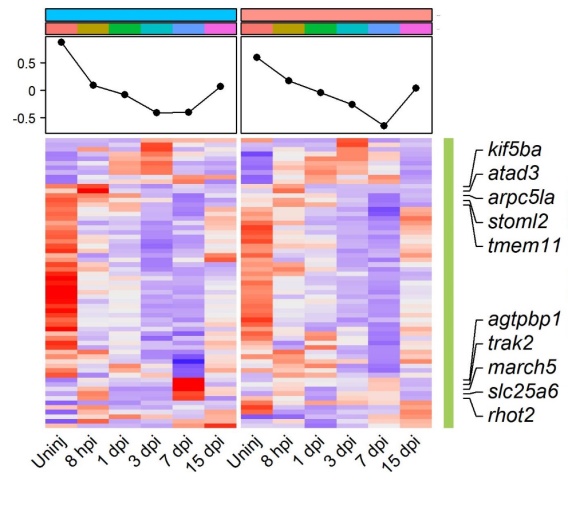

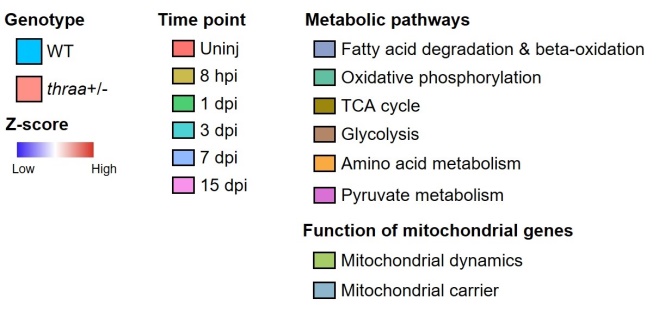

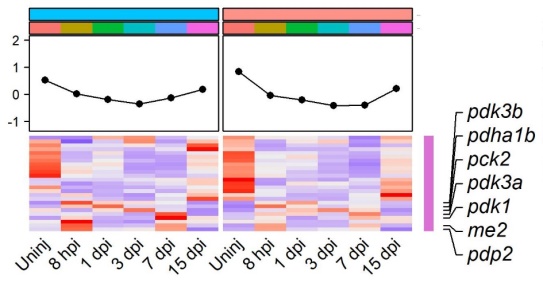

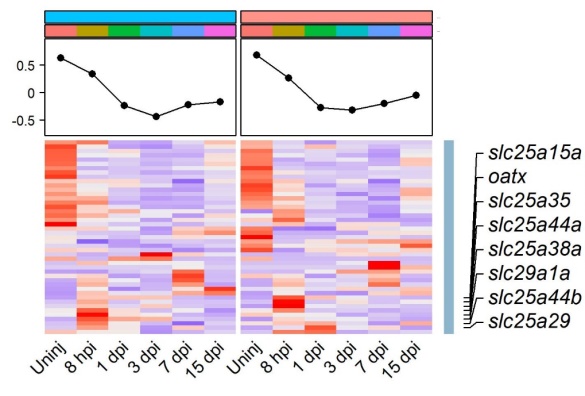

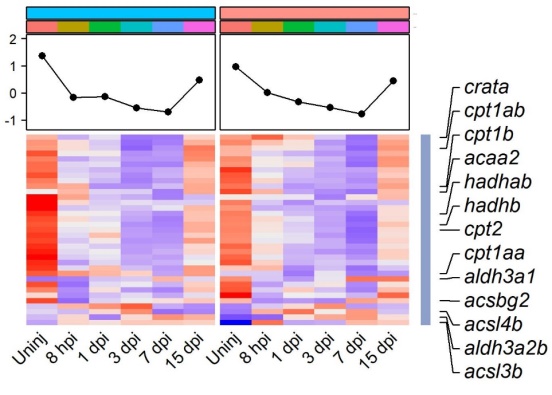


**B**

**D**

**C**

**E**

**G**

**F**

**H**

**A**

Supplementary Figure S7. The expression dynamics of metabolic and mitochondrial genes.

**A-H)** Heatmaps show the expression of genes in fatty acid degradation & beta oxidation (FAO) **A)**, oxidative phosphorylation (OXPHOS) **B)**, TCA cycle **C)**, glycolysis **D)**, amino acid metabolism **E)**, pyruvate metabolism **F)**, mitochondrial dynamics **G)**, and mitochondrial carrier **H)** as shown in Fig. 5A and 5B.

Supplementary Figure S8

**B**

**A**


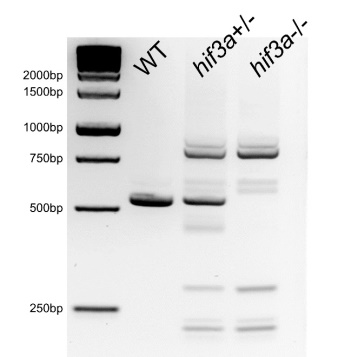

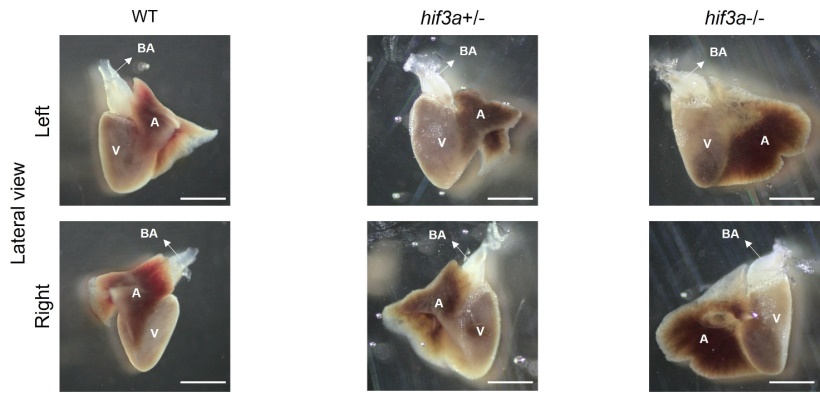

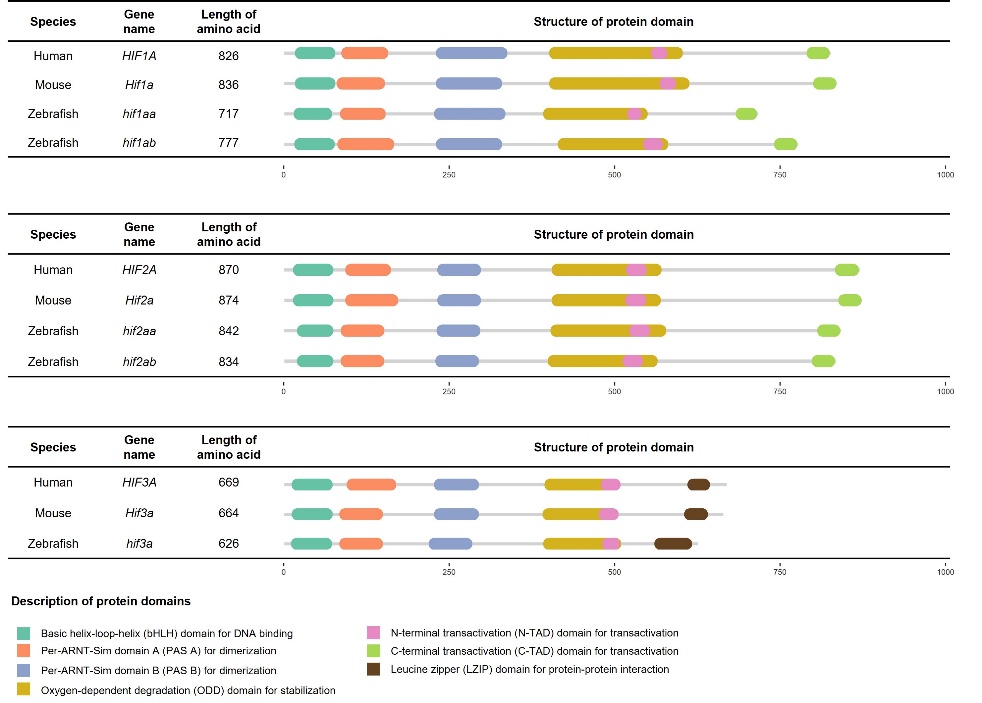

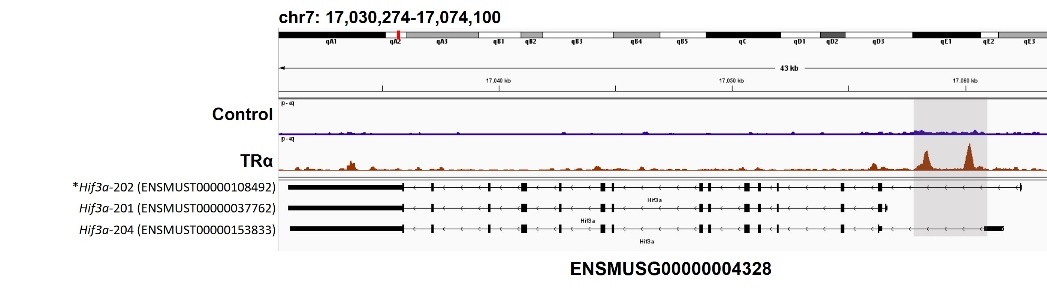

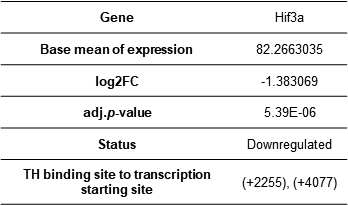

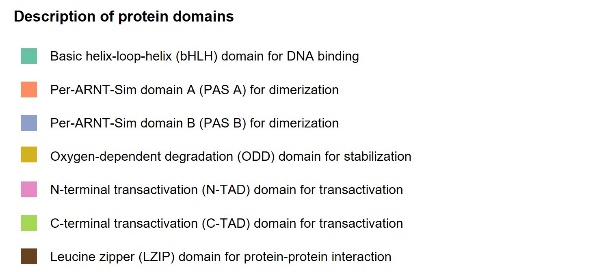

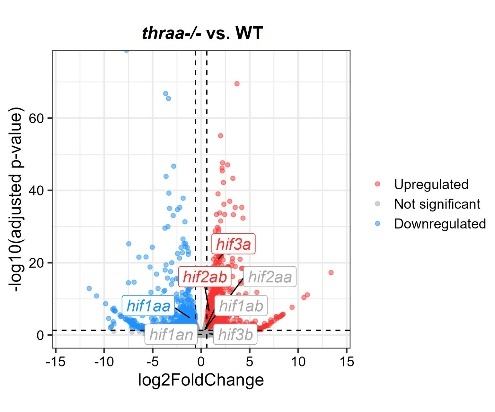

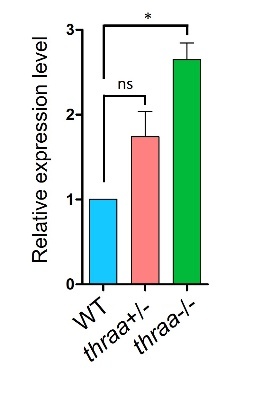

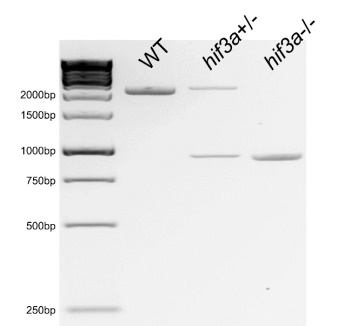


**G**

**H**

**I**

**F**

**C**

**D**

**E**

Supplementary Figure S8. Transcriptional regulation of HIFs and generation of *hif3a* KO model.

1. Volcan plot shows the DEGs in adult zebrafish heart by *thraa*^−/−^ mutants vs. WT. HIF genes are labelled in the plot.
2. RT-qPCR analysis of *hif3a* in adult zebrafish heart. *n* = 2 for WT, *thraa*^+/−^ mutants and *thraa*^−/−^ mutants. Results are shown in mean ± s.e.m; ns, *P* > 0.05; * *P* < 0.05. Statistical significance was calculated by two-tailed unpaired Student’s *t*-test.
3. Captured Integrated Genome Viewer (IGV) image of ChIP-Seq data illustrating the binding of TRα to *Hif3a* gene in mouse heart (*M. musculus*). ChIP-Seq data is obtained from GSE125414. Enriched binding region of TRα is highlighted in grey. *Hif3a*-202*: Ensembl canonical transcript. The table shows the detailed result of *Hif3a* in the ChIP-Seq data set.
4. Schematic diagram of protein domain structure of HIFα proteins in different species.
5. Representative image of genotyping results in *hif3a* mutant zebrafish using DNA. DNA spanning from exon 10 to exon 14 of *hif3a* was amplified. 1,127 bp is deleted in *hif3a* KO model. WT: 2,021 bp; *hif3a* mutant: 894 bp.
6. Representative image of genotyping results in *hif3a* mutant zebrafish using mRNA. Extracted mRNA spanning from exon 10 to exon 14 of *hif3a* was amplified. KO mutants show disrupted mRNA splicing in *hif3a* leading to mis-spliced transcripts.

**G-I)** Whole-mount images show the external appearance of adult WT **G)**, *hif3a*^+/−^ mutants **H)**, and *hif3a*^−/−^ mutants **I)**. A, atrium; V, ventricle; BA, bulbus arteriosus. Scale bar, 1 mm.
